# Species-Area Relationships Are Controlled by Species Traits

**DOI:** 10.1371/journal.pone.0037359

**Published:** 2012-05-21

**Authors:** Markus Franzén, Oliver Schweiger, Per-Eric Betzholtz

**Affiliations:** 1 Department of Community Ecology, UFZ, Helmholtz Centre for Environmental Research, Halle, Germany; 2 Division of Biodiversity, Department of Biology, Lund University, Lund, Sweden; 3 School of Natural Sciences, Linnaeus University, Kalmar, Sweden; University Copenhagen, Denmark

## Abstract

The species-area relationship (SAR) is one of the most thoroughly investigated empirical relationships in ecology. Two theories have been proposed to explain SARs: classical island biogeography theory and niche theory. Classical island biogeography theory considers the processes of persistence, extinction, and colonization, whereas niche theory focuses on species requirements, such as habitat and resource use. Recent studies have called for the unification of these two theories to better explain the underlying mechanisms that generates SARs. In this context, species traits that can be related to each theory seem promising. Here we analyzed the SARs of butterfly and moth assemblages on islands differing in size and isolation. We tested whether species traits modify the SAR and the response to isolation. In addition to the expected overall effects on the area, traits related to each of the two theories increased the model fit, from 69% up to 90%. Steeper slopes have been shown to have a particularly higher sensitivity to area, which was indicated by species with restricted range (slope  = 0.82), narrow dietary niche (slope  = 0.59), low abundance (slope  = 0.52), and low reproductive potential (slope  = 0.51). We concluded that considering species traits by analyzing SARs yields considerable potential for unifying island biogeography theory and niche theory, and that the systematic and predictable effects observed when considering traits can help to guide conservation and management actions.

## Introduction

The species-area relationship (SAR) is one of the best studied patterns in ecology, often being referred to as one of ecology’s few laws [1], [2]. Classical island biogeography theory predicts that species richness will increase with island area and decrease with isolation [3], [4]. It was developed on true islands, but has frequently been applied to a wide spectrum of island-like systems [5]. Despite its broad application, one of the objections raised includes the fact that classical island biogeography theory ignores functional differences among species and thus considers all species as ecologically equivalent, while relying on a dynamic equilibrium of colonization and extinction processes only [6], [7]. In contrast, niche theory focuses on the importance of environmental heterogeneity and the resultant niche partitioning as major drivers of species-richness patterns [3], [8]. It seems most likely that aspects covered by both theories act in combination to explain diversity patterns, suggesting the need for an integrated approach for a better understanding of SARs [9], [10].

There have been recent calls for such an integrative approach to include both deterministic and random components in order to enhance its predictive ability [10]. Classical island biogeography theory usually does not consider differences among species, but the relevant processes of colonization, persistence, and extinction are a combination of both stochastic and deterministic factors [11]. It is likely that the pure SAR may constitute a random aspect of an integrative approach, while allowing for differences among SARs according to species traits may constitute the deterministic part. These traits in turn may be related to the processes of persistence, colonization, and extinction, in addition to niche theory. Another integrative approach has been suggested recently by Sólymos and Lele [12]. They emphasized on the importance of understanding interactions among SAR parameters and modifying variables (species traits and area in our case) within a hierarchical modeling approach to make robust predictions. While Sólymos and Lele [12] focus on local variation, we investigate variation among trait states.

Extinction risks can be related to species traits such as trophic rank, reproductive capacity, and mobility [7], [13], [14]. The length of the flight period has often been used as a proxy for the reproductive potential in studies of insects and a longer adult activity is related to a larger number of offspring [15]. A large number of offspring may increase the survival probability of populations in small areas, since it enhances the chances of colonization, successful population establishment, and population recovery [16]. Population persistence can be affected by population size, range size, or other measures of rarity. Rare or range-restricted species, or species with small average population sizes, may be absent from small or isolated islands because of a reduced ability to colonize otherwise suitable areas [17], [18]; alternatively such species may suffer a high extinction risk, because of their often small local populations [7], [19], [20]. Further specialization can be assumed to increase the extinction risk. Diet and habitat generalists can utilize more resources and take advantage of ephemeral habitats [7], [21]. Specialized species may be more sensitive to environmental change [20], i.e. from extreme weather situations, parasitoids, or diseases [22], resulting in an increased extinction risk.

Body size has often been used as a proxy for mobility in studies of insects and a larger size may increase the persistence of populations in small and isolated areas because of an expected high mobility. However, the relationship between mobility and body size often seems to be rather weak or statistically insignificant [23], [24]. In contrast, the opposite may also be true, since larger species have higher energy needs and larger area requirements, which could reduce their persistence on small islands.

Surprisingly few empirical studies have explicitly addressed whether species with contrasting traits differ in their SARs; in this respect, there seem to be more studies from fragmented habitats than from true islands [25], [26]. In this study, we focused on butterflies and moths on true islands. No quantitative analyses have previously been conducted with data from true islands to investigate whether traits are related both to processes of colonization and extinction (with respect to island biogeography theory), and to niche theory (specific species responses to area and isolation). Here we explored the slope of the SARs in combination with island isolation, using data from eight true islands and the following eight species traits: reproductive potential, abundance, range size, temporal population trend, body size, adult activity temperature, larval dietary breadth, and habitat niche.

We tested the following predictions:

1. Species richness increases with area and decreases with isolation.
2. Species traits relevant for the processes of colonization, persistence and extinction and for niche theory contribute to modulating the overall effects of SARs.

## Results

Based on a total of 1016 butterfly and moth species, we found the expected positive relationship between overall species richness and area (Table 1). Although the slopes of the SARs differed among the taxonomic groups, the general patterns remained constant (Table 1). The explanatory power of area was high (coefficient of determination based on deviance, D^2^ = 69%). However when species traits were included in the models, the explained variation increased to 78% for population trend and up to 90% for range size (Table 1).

**Table 1:** The best-fitting (lowest AICc) generalized linear mixed effects models for the relationship between species richness and area for all species, for different taxonomic groups and for eight traits considered separately.

Trait	D^2^ (%)	Trait state	Intercept	Slope SAR	P-value	Significance between trait states
Total	69	overall	3.95	0.23	<0.001	
Reproductive potential	83	low	1.29	0.51	<0.001	l-m
		moderate	2.02	0.44	<0.001	m-h
		high^a^	3.13	0.25	<0.001*	h-m, h-l
Abundance	87	low	2.10	0.52	0.007	l-m, l-h
		moderate	1.90	0.23	0.670	m-l
		high^a^	2.44	0.20	<0.001*	h-l
Range size	90	small	−0.73	0.82	<0.001	s-m, s-l
		moderate	2.00	0.50	<0.001	m-s, m-l
		large^a^	3.32	0.26	<0.001*	l-m, l-s
Population trend	78	decreasing^a^	1.79	0.47	<0.001*	d-i, d-s
		increasing	2.59	0.27	<0.001	i-d
		stable	2.68	0.26	<0.001	s-d
Body size					ns	
Adult activity temperature	83	cold^a^	1.92	0.48	<0.001*	c-w
		warm	3.30	0.27	<0.001	w-c
Habitat niche	80	forest^a^	1.85	0.39	<0.001*	f-g
		open	2.29	0.36	0.527	o-g
		generalist	2.85	0.25	0.023	g-f, g-o
Larval dietary breadth	83	specialist	0.62	0.59	<0.001	s-o, s-g
		oligolect	2.53	0.35	0.009	o-s, o-g
		generalist^a^	2.72	0.25	<0.001*	g-o, g-s
Total [Taxonomic group]	NA	butterflies	3.01	0.37		
		Geometridae	5.00	0.25		
		Pyralidae	4.35	0.24		
		others	3.95	0.23		
		Sphingidae	1.96	0.17		
		Noctuidae	5.42	0.15		

Taxonomic group was included as a random factor to control for possible taxonomic dependence. When the interaction of area and trait was significant at P<0.05, separate slopes for each trait state are provided and tests (P-values) for the deviation of the SAR slopes from zero are given for the initial reference trait state. Significant pairwise relationships between trait states, based on changed contrasts, are presented by the first letter of the trait states, e.g. significant difference between low and moderate is indicated by l-m. The trait states are sorted by decreasing SAR slopes. D^2^, deviance-ratio based on the coefficient of determination (pseudo R^2^); D^2^ for taxonomic group was not available (NA) because taxonomic group was included as a random factor. ns  =  not significant.

a – reference trait states for which P-values for test of differences from zero are provided.

**Figure 1:**
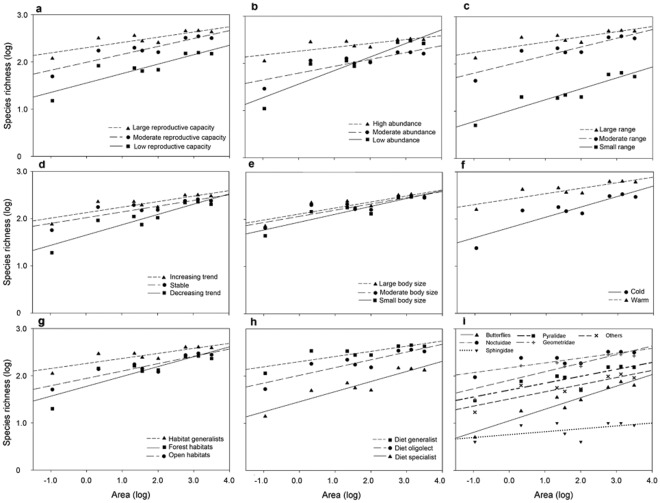
Species-area relationship for eight different traits and their states: a) reproductive potential; b) abundance; c) range size; d) population trend; e) body size; f); adult activity temperature; g) habitat niche; h) larval dietary breadth; and i) taxonomic group.

The slopes of the SARs differed significantly among states for all traits except body size (Fig. 1; Table 1). With respect to rarity, species with small ranges, average low abundances, and a declining population trend were most sensitive to area. In particular, the slopes of the SARs were significantly steeper for species with low reproductive capacity and species adapted to lower temperatures. Furthermore, specialist species with respect to larval dietary breadth and habitat were associated with a steeper SAR slope (Fig. 1; Table 1).

## Discussion

### Traits Related to Both Island Biogeography and Niche Theory Define SARs

It is possible to assume stochastic processes of classical island biogeography by calculating SARs, independent of any species traits and thus solely based on the richness of the different taxonomic groups; in this way, it is possible to obtain a fair amount of explanatory power (69%). Although the slopes of the SARs varied among the different taxonomic groups, the expected pattern of increasing species richness with increasing size of the islands emerged. However, when we additionally considered more deterministic effects by allowing the slopes of the SARs to vary according to different states of species traits; the explanatory power of the SAR increased considerably (up to 90%). We found that traits associated with niche theory, i.e. dietary and habitat niche breadth, were as important as traits related to colonization and extinction processes (with respect to island biogeography). Therefore, considering species traits provides considerable potential for improving the assumptions of island biogeography and for a unification with niche theories [9]. Thus, by advancing the assumption of classical island biogeography of simple area-dependent colonization and extinction rates [3], [27] to more deterministic trait-mediated area-dependencies, SARs can improve our understanding not only of patterns in species richness but also of different levels of vulnerabilities and consequent systematic changes in species compositions. Such an improved understanding of systematic changes, in addition to stochastic components, can allow further inferences covering a much broader spectrum of biodiversity patterns such as gradients in endemicity and rarity, evolutionary processes on islands, and effects on ecosystem functioning and ecosystem services [4], [28].

Our approach is in accordance with recent suggestions on how to deepen our understanding of SARs by using models that allow for the inclusion of multiple focal parameters (intercept, slope, specific sources of variability) [12]. While Sólymos and Lele [12] investigated how to include local variation of SARs, we included the variation among trait states as modifying covariates, using a similar hierarchical approach with mixed effects models (trait and family). Such an integrated, generalised approach clearly has great potential and increases the predictive power of SARs (Table 1). It can also serve as a strong tool for applied ecology, especially when predictions should be made for cases or areas with no or sparse background data. Including species traits will also provide a better mechanistic understanding of the modifiers of the SAR patterns and can thus help to improve decision making in conservation.

### The Slopes of the SARs for Island Communities are Steeper Compared to Mainland Communities

Surprisingly, our study is among the first that explores trait-dependencies of SARs using data from true islands [7]. Studies of island communities have advantages over those from fragmented mainland populations, because they are free of confounding matrix effects and the definition of the borders is clearer than for terrestrial habitat patches [29]. The absence of any effects of a surrounding matrix leads to the expectation that slopes of the SAR will be steeper on islands, which was met by our results [30], [31]. Even though comparisons of slopes among SAR studies might be biased depending on how the study was performed a comparison is interesting to put our study in the perspective to others. The overall slope of the SAR for the analysed lepidoptera was 0.23, ranging from 0.15 (Noctuidae) to 0.37 (butterflies). These values are well within the ranges of those reported from other studies on true islands [32], [33]. Examples of slopes found previously are: 0.10 for woody plants [34], 0.16 for land snails [34], 0.32 for plants from the Galapagos [32], 0.34 for beetles in the West Indies [35], 0.36 for carabid beetles [34], and 0.62 for forest birds [34]. Among butterflies and moths, slopes of 0.14 have been found for Sphingidae in the Malaysian archipelago [36], of 0.20 for butterflies of the West Indies [37], and 0.67 for butterflies from islands in the Baltic sea [38]. However, the observed slopes of the SARs, especially those of the butterflies, were steeper than reported from studies of butterflies in terrestrial habitat patches, e.g. slopes of 0.15 for the Rocky Mountains [39], 0.10 for Northern and Eastern European countries [40], 0.16 for calcareous grasslands in Germany [41], and 0.12 according to a meta-analysis of moths and butterflies across several countries in Europe and North America [15].

When the SARs were allowed to vary according to the trait states, larger ranges were evident for the slopes: from 0.20 for species with high abundance to 0.86 for species with a small range size. Nevertheless, the observed slopes were still steeper than those of comparable groups from mainland habitats. Öckinger *et al*. [15] reported slopes of 0.22 and 0.15 for specialist species with low reproduction and for generalist species with high reproduction, respectively, while we found comparable values of 0.51 and 0.25 for species with low and high reproduction (Table 1). Steffan-Dewenter and Tscharntke [25] found an increasing trend of the slopes for polyphagous (0.07), strongly oligophagous (0.16), and monophagous species (0.22), while the comparable values from our study are 0.25, 0.35, and 0.59 (Table 1). Krauss *et al*. [41] showed differences for habitat specialists (0.40) and generalists (0.10), but at least the generalist species from our study had a considerably steeper slope (specialists: 0.39, generalists: 0.25). The rather shallow slopes reported for butterflies from habitat fragments are likely to be an effect of the matrix [42]. Since the matrix surrounding terrestrial habitat patches is usually not uniformly hostile, it can provide some buffer capacities against extinctions. Animals venturing outside patches may find sufficiently benign conditions to live and reproduce, at least for a short time, rendering the notion of the patch less relevant [31].

### Traits Related to Island Biogeography Theory: Colonization, Persistence and Extinction

The high sensitivity of species with low reproductive potential to island area might be explained by a decreased potential of such species to recover rapidly from population collapses, which can be important on small islands where environmental stochasticity is likely to be high [16]. Furthermore, species with low reproductive potential can also produce fewer potential colonizers, resulting in a lower probability of re-colonization after local population extinctions [43].

Rarity can be related to the species traits: abundance, range size, and temporal population trend [44]. Species with high abundance, large ranges, and with stable or increasing trends were less affected by changes in area (Fig. 1). This is consistent with findings that low densities, restricted ranges, and negative population trends–often associated with rare species–predispose species to extinction [18]. The presence of a large number of individuals can prevent extinctions by limiting population collapses and enabling rapid re-colonization. Abundant species are generally less sensitive to changes in area [7], [45], and the potential for sea crossings seems to be related to the abundance of the species [46].

Species active during the warmest period of the year (daily mean temperature >16°C) were less sensitive to island area (Fig. 1). However, in the context of climate warming this could mean that increased temperatures may increase the mobility of some butterfly and moth species, which would in turn make them less sensitive to changes in area. Climate warming has already prolonged the activity period and caused increased voltinism [47]. Our results suggest that species able to adapt to climate warming by having an increased number of generations might benefit from both an increased reproductive potential and increased mobility, making them less sensitive to changes in area.

**Figure 2:**
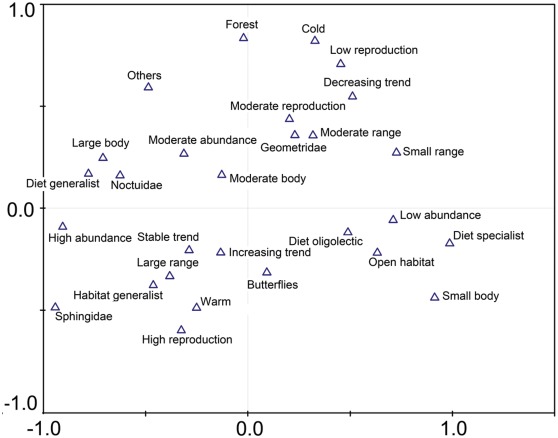
Correspondence analysis (CA) showing the relationships among the analysed trait states and taxonomic groups. Pyralidae were not included in the analysis because there were no data for abundance and population trend for this taxonomic group.

We could not identify any effect of body size on the SAR (Fig. 2). Large body size may increase the persistence of populations in small and isolated areas because of an expected high mobility. However, the opposite may also be true, since larger species have larger energy and area requirements [48], which could reduce the persistence and increase the extinction risk of local populations of large-bodied species on small islands. This in turn would reduce the positive effect of a potentially higher mobility among large species [24]. Another study explored species richness patterns of bees and also found no clear effect of body size on the SAR [49].

### Traits Related to Niche Theory: Habitat Specialization and Dietary Specialization

We observed that sensitivity to changes in area increases from generalists to specialists with respect to both larval diet and habitat use [25], [41]. Species with a wider ecological tolerance in their diet and habitat are more likely to find suitable host plants and habitats [7], [50]. Thus, they may experience increased colonization success and may be able to develop sustainable populations when resources are limited in small areas [51].

High and synchronized population variability among specialists can increase their extinction risk, especially in small areas where resources are limited [52]. In contrast, the potential to use several host plants and habitats can ensure population survival by providing a broader range of micro-sites. Indeed, diet specialists often occupy relatively small proportions of the ranges of the host they consume [53]. This might be because they are more sensitive to changes in area than are generalists. Although we used broad classes of habitat niche to explore whether generalists differ in their SARs from specialists, our results indicate that traits related to niche theory perfectly complement traits usually associated with colonization and extinction and can help to improve our understanding of the underlying mechanisms of SARs.

The multivariate analysis showed that traits are often interlinked with each other. For instance, wide ranging species are predominantly active at warmer temperatures, and thus can be considered to be more mobile [23], [54], [55]; but they are also often habitat generalists (Fig. 2). A potentially greater mobility and the larger number of utilized habitats in combination can in turn increase a species’ ability to persist on smaller islands. Further, it seems that body size is linked to dietary breadth and abundance (large body size, high abundance, generalists, Fig. 2). Thus we recommend that body size is considered more appropriately as a proxy for resource use than for mobility, which is consistent with other studies suggesting that dietary specialists are less mobile than dietary generalists [24], [56], [57].

### No Significant Effect of Isolation

We found no effect of isolation. In most studies the effect of isolation is very weak and a meta-analysis by Prugh *et al*. [31] did not show any interactive effects of traits and connectivity across species in terrestrial habitats, and isolation generally seems to play a minor role in mainland areas and less isolated islands (<4 km from the mainland) [34]. Our result suggest that 90 km is not enough to detect any isolation effects, but given the low power of our analysis (because of the restricted number of islands investigated), such a non-significant result needs to be taken with great caution. However, given the low statistical power in our study, we are nevertheless confident that the observed effects of how traits modify the SAR are robust and general and in fact they are well supported by theory.

Another critical point related to the restricted number of investigated islands might be potential confounding effects of land use. The small number of data points might influence our results, especially when systematic effects occur, e.g. when the small islands are more intensively used than the large ones or vice versa. However, when comparing the islands it is evident that land use has been, and still is, comparably similar among the islands. Only the size and distribution of the resources (habitats and host plants) differ according to the size of the islands. However, these differences in patch size, quality and distribution can be regarded as an effect mainly related to island size, and not to a potential bias by human land use.

### Conclusions

Here we show that considering species traits is a promising way to add deterministic effects to the stochastic and neutral nature of island biogeography theory. Moreover, traits are relevant for processes of colonization and extinction with respect to island biogeography theory. Similarly, traits relevant for niche theory modulate overall SARs well. Hence there is a need to unify the two theories [9], and this study highlights that species traits are important to consider. Including covariates and interactions when exploring SAR models has recently been developed [12]. By shifting the focus from simple area-dependent colonization and extinction rates to more deterministic trait-mediated area-dependencies, such a unified approach can substantially improve our understanding of biodiversity patterns beyond that of species richness. Furthermore, a better understanding of SARs will provide new insights, such as the calculation of extinction depths [4], the assessment of systematic shifts in the composition of communities in the course of global change; this will improve prediction ability and decision making in conservation. In particular, our results indicate that species with small range sizes, species with low local abundances and diet specialists are particularly sensitive to changes in area. In contrast, common, highly mobile generalists with large ranges and species active in warm temperatures are less sensitive to area. An increasing dominance of these species over rare, sedentary specialists could however have profound implications for ecosystem functioning and might lead to cascading effects at higher and lower trophic levels.

## Materials and Methods

### Data Sets

**Figure 3:**
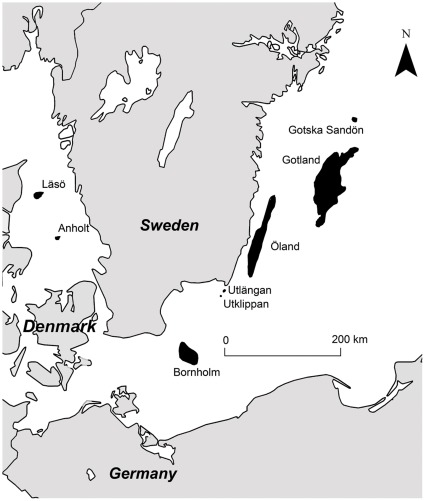
Locations of the eight studied islands (black areas).

We searched the literature for distribution checklists, and used personal contacts to collect data sets of Lepidoptera on true islands (landmasses surrounded by water). To rule out possible effects of climate and geography [58], we restricted our search to the 54–58° N latitudinal range, and to the Baltic sea. We found eight islands where data quality was sufficient for further analysis (Table 2, Fig. 3). For these eight islands, moths have been studied extensively including whole season surveys using light-traps. Butterfly data were collected by at least six surveys. All records from each island until 2008 were included in the analyses. The intensive surveys on these islands ensure almost complete species lists for a comparable time period, which is reflected by very low numbers of new species in the last two years (Table 2).

**Table 2:** Characteristics of the eight studied islands arranged by decreasing island area.

Island	Country	Longitude N	Latitude E	Area (km^2^)	Distance to mainland (km)	Number of Lepidoptera species^1^	Number of new species during 2009 and 2010	Source^2^
Gotland	S	57° 29.631′	18° 33.627′	3140	87	896	4	[63]
Öland	S	56° 43.818′	16° 42.885′	1342	3.5	961	3	[63]
Bornholm	DK	55° 6.316′	14° 53.461′	588	36	925	5	[79]
Läsö	DK	57° 14.550′	11° 02.039′	101	18	483	NA	Nielsen unpubl. data
Gotska Sandön	S	58° 21.780′	19° 14.826′	36	90	512	2	[63]
Anholt	DK	56° 42.286′	11° 34.477′	22	45	632	NA	[80]
Utlängan	S	56° 1.235′	15° 47.369′	2	5	573	6	Betzholtz unpubl. data
Utklippan	S	55° 57.271′	15° 42.220′	0.1	16	182	NA	Betzholtz unpubl. data

1 Includes all Macrolepidoptera and the additional families Hepialidae, Cossidae, Zygaenidae, and Pyralidae.

2 Sources are updated and includes all records to 31 December 2008 from respective island.

Since the number of islands used for analyses (eight) is quite low, we are aware that the resulting statistical power might be low, giving rise to an increased probability of type II errors and the consequent inability to detect ecological relationships. However, we can be confident that the probability of type I errors, which may lead to falsely inferred relationships, is also quite low. As a consequence, we can draw strong conclusions on the basis of significant relationships, while non-significant relationships should be interpreted with greater caution.

We excluded all species that do not reproduce in the study area, because their appearance is irruptive and strongly correlates with search time and specific weather conditions [59]. For each island, we used the observed identities and species richness. We extracted trait data from the literature for all species, and restricted our analyses to the best known taxonomic groups of Lepidoptera, including butterflies, macro-moths, and the additional families Hepialidae, Cossidae, Zygaenidae, and Pyralidae (Table S1).

### Description of the Islands Studied

#### Gotland

Gotland is the largest island in the Baltic Sea, located approximately 90 km east of the Swedish mainland, and about 130 km from the Baltic States. Gotland is composed of lime rocks, and has mixed habitats with arable fields, pastures, forests, and shallow lakes. The island area is 2994 km^2^ and there are 57,200 residents.

#### Öland

Öland is the second largest Baltic island, located approximately 6 km east of the Swedish mainland. The island is on a limestone plateau. Öland is dominated by agricultural landscape, but there are also coastal meadows, wetland areas, alvar land, and deciduous and coniferous forests. The dominant environmental feature of the island is the Stora Alvaret, a limestone pavement that is the habitat of numerous rare and endangered species. The island area is 1342 km^2^ and there are 25,000 residents.

#### Bornholm

Bornholm is a Danish island in the Baltic Sea, located 15 km east of the Swedish coast. The topography of the island consists of dramatic rock formations in the north, sloping down towards areas of pine and deciduous forests and farmland in the middle parts, and sandy beaches in the southern parts. The island area is 588 km^2^ and there are 42,200 residents.

#### Læsø

Læsø is the largest island in the North Sea bay of Kattegat, and is located 19 km northeast of the Danish mainland. The island was deforested approximately 100 years ago, and is dominated by open and dry habitats. The island area is 114 km^2^ and there are 2,000 residents.

#### Gotska Sandön

Gotska Sandön is a Swedish island in the Baltic Sea, located 40 km north of the Baltic island Gotland and 90 km east of the Swedish mainland. Gotska Sandön is mostly a sand island, the landscape is dominated by beaches, dunes, and pine forests. Only small patches of the island are colonized by deciduous forest, shrub, and grassland habitat. The island area is 36 km^2^ and there are no permanent residents.

#### Anholt

Anholt is a Danish island in the North Sea bay of Kattegat, located 45 km west of the Swedish west coast. The western part of Anholt is a moraine landscape. The eastern part of the island consists of dry and open habitats dominated by lichen heaths. The island area is 22 km^2^ and has a population of 170 residents.

#### Utlängan

Utlängan is an island in the Baltic Sea, located 8 km south of the Swedish mainland. Utlängan consists of woodlands and meadows. The island area is 2.15 km^2^ and there is only one permanent resident.

#### Utklippan

Utklippan is a remote island in the Baltic Sea, located 16 km south of the Swedish mainland. The vegetation on Utklippan is very sparse, and is restricted to crevices in the rocks, with only a few isolated bushes and trees. The island area is 0.09 km^2^ and there are no permanent residents.

### Definitions of the Analysed Traits

#### Reproductive potential

We used the average length of the flight period in weeks in southern Sweden as a proxy for the reproductive potential of each species. Reproduction is strongly related to the adult life-span of a species [15]. For species with two generations per year, we summed the flight periods. We classified the length of the flight period into the following categories: short (2–4 weeks, n = 173); moderate (5–6 weeks, n 375); and long (7–20 weeks, n = 476).

#### Abundance

We used abundance data for moths from Denmark [60] and Sweden (unpublished data). In Sweden, data from light-traps at 13 localities along the coast of the Swedish mainland, which included three light-traps on the studied islands, were used. The light-traps were running for at least one year between 2003 and 2008. For butterflies and other diurnal species, we used data from transect surveys in southern Sweden covering 170 localities (unpublished data). Abundance was measured as the number of individuals recorded per year, and classified as low (0–30 individuals, n = 341), moderate (31–99 individuals, n = 181), or high (100–4467 individuals, n = 334). It was not possible to generate the required data for the family Pyralidae.

#### Range size

We determined the number of European countries in which the species have been recorded according to Karsholt and Razowski [61]. We used the number of European countries because this is the most homogenous dataset available across all taxonomic groups of butterflies and moths. Due to the fact that we used the number of species sharing a certain trait as the dependent variable in our analyses, we did not consider range size as a continuous variable in the model. Instead, we classified species as having a small (5–19 countries, n = 87), moderate (20–27 countries, n = 420), or large (28–36 countries, n = 509) range size.

#### Population trend

The population trend of each species in the region was defined in the analysis as being stable (n = 264), increasing (n = 326), or decreasing (n = 266). The three categories are based on data on earlier distributions [62], as well as data from unpublished recent surveys, provincial records [63], and yearly reports [64]. Declining species were defined as those whose distribution area substantially reduced during the last 50 years (i.e. they became extinct in at least one province in Sweden). The species that had increased their range were defined as those whose range substantially expanded during the last 50 years (i.e. they colonized at least one province over this period). The other species were classified as stable. It was not possible to generate the required data for the family Pyralidae.

#### Body size

We collected data on wingspan (mm) from the literature [65]–[71]. Because we used the number of species sharing a certain trait as the dependent variable in our analyses, we did not consider wingspan as a continuous variable in the model. Instead we classified species as having a small (11–25 mm, n = 317), moderate (26–35 mm, n = 345), or large (36–105 mm, n = 354) wingspan. In another study, small butterflies and moths were defined as having a wingspan less than 32 mm and large ones as having a wingspan greater than 32 mm [15].

#### Adult activity temperature

We categorized species according to the mean daytime temperature during the adult activity period [72]. Species where the mean daytime temperature of the adult activity period was above 16°C were classified as ‘warm’ species (n = 650). Species active during other periods of the year were classified as ‘cold’ species (n = 366). In the study area, the period for ‘warm’ species normally ranges from 20th July to 10th September [73].

#### Habitat niche

Each species was classified according to its preferred habitat using the following three classes: species from open habitats (grasslands, wetlands, and other open areas including shrub and brushwood habitats, n = 321), species from forest habitats (n = 279), habitat generalists (species occurring in all habitats, n = 416). The information on habitat preferences was extracted from the literature [65]–[71].

#### Larval dietary breadth

We classified the larval dietary breadth into three classes: specialist species that feed mainly on a single plant species (n = 170), oligophagous species that feed on a few plant species (less than six or restricted to a particular plant genus/family; n = 393), and generalist species that feed on several different plant species (six or more) or genera (n = 453). Information about food plants was extracted from the literature [65]–[71].

#### Taxonomic group

Taxonomic group was included as a random factor in the analysis to control for a possible bias of taxonomy, since SARs may differ according to obvious morphological differences [74]. For example, Sphingidae are dominated by large, robust, mobile, night-active species, while butterflies are dominated by diurnal, sun-dependent, and often more fragile species. We used the following categories: butterflies (n = 80), Geometridae (n = 309), Noctuidae (n = 344), Pyralidae (n = 160), Sphingidae (n = 10), and ‘other macro-moths’ (n = 113). ‘Other macro-moths’ included the families: Arctiidae, Cossidae, Endromidae, Hepialidae, Lasiocampidae, Limacodidae, Lymantriidae, Nolidae, Notodontidae, Saturniidae, and Zygaenidae. Families were pooled in the case of ‘butterflies’ and ‘other macro-moths’ to avoid small numbers in some families (Table S1).

### Statistical Analyses

To assess a baseline relationship independent of any species traits, we related overall richness of butterflies and moths to the log-transformed area and isolation (measured as the shortest Euclidean distance from the edge of the island to the mainland) and their interaction term using a generalized linear mixed effects model with a Poisson error distribution, the log-link function, and treatment contrasts. To account for different potential responses of the different taxonomic groups, we allowed random intercepts and random slopes for each taxonomic group. We controlled for over-dispersion by accounting for individual-level variability in the random structure [75]. Since isolation was not significant in this baseline model, we did not consider it useful for the subsequent analysis, in which we developed generalized linear mixed effects models, as described above, separately for each trait. We related species richness to area, trait state, and their interactions. As in the baseline model, we included taxonomic group as a random effect, and allowed for random slopes of the SAR for each trait state. In doing so, we avoided problems of pseudo-replications, indicative of the calculated species richness per trait state and family. We also tested for interactions between area and trait states. Once a significant interaction was found, we systematically tested for pairwise differences by modifying the contrasts (i.e. by changing the trait state against which the other states are tested). Model selection was based on minimizing the second order Akaike Information Criterion (AICc). After selecting the combination of random effects according to AICc, a hierarchical model selection for the fixed effects was conducted to determine the most parsimonious combination of fixed and random effects [76]. Since some states of different traits tend to be linked (e.g. a broad habitat niche and large range sizes or small dietary niche breadth and low reproduction [77], [78]), we explored the relationship between the analyzed trait states across the species using correspondence analysis (CA) in which all trait states were dummy-coded. Since data were not available for abundance and population trend for the Pyralidae, they were excluded from the CA. All models were developed using the lme4 package in the R software environment (R development Core Team version 2.13.2, 2011). The multivariate CA was performed in Canoco ver 4.5.

## Supporting Information

Table S1 Scientific names, the number of the eight islands where the species has been recorded and their taxonomic group. The list is sorted systematically according to Karsholt and Razowski [61].
(DOCX)

## References

[1] Arrhenius A (1921). Species and area.. J Ecol.

[2] Rosenzweig ML (1995). Species diversity in space and time..

[3] MacArthur RH, Wilson EO (1967). The theory of island biogeography..

[4] Triantis KA, Borges PAV, Ladle RJ, Hortal J, Cardoso P (2010). Extinction debt on oceanic islands.. Ecography.

[5] Baz A, GarciaBoyero A (1995). The effects of forest fragmentation on butterfly communities in central Spain.. J Biogeogr.

[6] Hubbell SP (2001). The unified neutral theory of biodiversity and biogeography..

[7] Holt RD, Lawton JH, Polis GA, Martinez ND (1999). Trophic rank and the species-area relationship.. Ecology.

[8] Hortal J, Triantis KA, Meiri S, Thebault E, Sfenthourakis S (2009). Island species richness increases with habitat diversity.. Am Nat.

[9] Kadmon R, Allouche O (2007). Integrating the effects of area, isolation, and habitat heterogeneity on species diversity: A unification of island biogeography and niche theory.. Am Nat.

[10] Lomolino MV, Brown JH (2009). The reticulating phylogeny of Island biogeography theory.. Q Rev Biol.

[11] Nekola J, Brown JH (2007). The wealth of species: ecological communities, complex systems, and the legacy of Frank Preston.. Ecol Letters.

[12] Sólymos P, Lele SR (2012). Global pattern and local variation in species–area relationships.. Global Ecol Biogeogr.

[13] Kotiaho J, Kaitala V, Komonen A, Päivinen J (2005). Predicting the risk of extinction from shared ecological characteristics.. Proc Natl Acad Sci U S A.

[14] Franzén M, Johannesson M (2007). Predicting extinction risk of butterflies and moths (Macrolepidoptera) from distribution patterns and species characteristics.. J Insect Conserv.

[15] Öckinger E, Schweiger O, Crist TO, Debinski DM, Krauss J (2010). Life-history traits predict species responses to habitat area and isolation: a cross-continental synthesis.. Ecol Letters.

[16] Henle K, Davies KF, Kleyer M, Margules C, Settele J (2004). Predictors of species sensitivity to fragmentation.. Biodiv Conserv.

[17] Taylor LR, Woiwod IP (1980). Temporal stability as a density dependent species characteristic.. J Anim Ecol.

[18] Mace GM, Kershaw M, Kunin WE, Gaston KJ (1997). Extinction risk and rarity on an ecological timescale.. The Biology of rarity: the causes and consequences of rare-common differences.

[19] Gaston KJ, Blackburn TM, Greenwood JJD, Gregory RD, Quinn RM (2000). Abundance-occupancy relationships.. J Appl Ecol.

[20] Pimm SL, Lee HJ, Diamond J (1988). On the risk of extinction.. Am Nat.

[21] Inkinen P (1994). Distribution and abundance in British noctuid moths revisited.. Ann Zool Fenn.

[22] Thomas JA (2005). Monitoring change in the abundance and distribution of insects using butterflies and other indicator groups.. Philos T Roy Soc B.

[23] Betzholtz PE, Franzén M (2011). Mobility is related to species traits in noctuid moths.. Ecol Ent.

[24] Sekar S (2012). A meta-analysis of the traits affecting dispersal ability in butterflies: can wingspan be used as a proxy?. J Anim Ecol.

[25] Steffan-Dewenter I, Tscharntke T (2000). Butterfly community structure in fragmented habitats.. Ecol Letters.

[26] Cagnolo L, Valladares G, Salvo A, Cabido M, Zak M (2009). Habitat fragmentation and species loss across three interacting trophic levels: effects of life-history and food-web traits.. Conserv Biol.

[27] Lomolino MV, Riddle BR, Whittaker RJ (2010). Biogeography, fourth edition..

[28] Triantis KA, Mylonas M, Lika K, Vardinoyannis K (2003). A model for the species-area-habitat relationship.. J Biogeogr.

[29] Dennis RLH, Shreeve TG, van Dyck H (2003). Towards a functional resource-based concept for habitat: a butterfly biology viewpoint.. Oikos.

[30] Brotons L, Monkkonen M, Martin JL (2003). Are fragments islands? Landscape context and density-area relationships in boreal forest birds.. Am Nat.

[31] Prugh LR, Hodges KE, Sinclair ARE, Brashares JS (2008). Effect of habitat area and isolation on fragmented animal populations.. Proc Natl Acad Sci U S A.

[32] Preston FW (1962). The canonical distribution of commonness and rarity: part I. Ecology. http://dx.doi.org/10.2307/1931976.

[33] Drakare S, Lennon JJ, Hillebrand H (2006). The imprint of the geographical, evolutionary and ecological context on species-area relationships.. Ecol Letters.

[34] Nilsson SG, Bengtsson J, Ås S (1988). Habitat diversity or area per se? Species richness of woody plants, carabid beetles and land snails on islands.. J Anim Ecol.

[35] Darlington PJ (1943). Carabidae of mountains and islands: data on the evolution of isolated faunas, and on atrophy of wings.. Ecol Monogr.

[36] Beck J, Kitching IJ, Linsenmair KE (2006). Determinants of regional species richness: an empirical analysis of the number of hawkmoth species (Lepidoptera : Sphingidae) on the Malesian archipelago.. J Biogeogr.

[37] Davies N, Smith DS (1998). Munroe revisited: A survey of West Indian butterfly faunas and their species-area relationship.. Global Ecol Biogeogr.

[38] Itämies J (1983). Factors contributing to the succession of plants and Lepidoptera on the islands off Rauma, Sw Finland..

[39] Wilcox BA, Murphy DD, Ehrlich PR, Austin GT (1986). Insular biogeography of the montane butterfly faunas in the Great Basin: comparison with birds and mammals.. Oecologia.

[40] Ulrich W, Buszko J (2003). Species-area relationships of butterflies in Europe and species richness forecasting.. Ecography.

[41] Krauss J, Steffan-Dewenter I, Tscharntke T (2003). How does landscape context contribute to effects of habitat fragmentation on diversity and population density of butterflies?. J Biogeogr.

[42] Öckinger E, Bergman K-O, Franzén M, Kadlec T, Krauss J (2012). The landscape matrix modifies the effect of habitat fragmentation in grassland butterflies.. Landscape Ecol.

[43] Brown JH, Kodric-Brown A (1977). Turnover rates in insular biogeography: effect of immigration on extinction.. Ecology.

[44] Brown JH (1984). On the relationship between abundance and distribution of species.. Am Nat.

[45] Lennon JJ, Koleff P, Greenwood JJ, Gaston KJ (2004). Contribution of rarity and commonness to patterns of species richness.. Ecol Letters.

[46] Dennis RLH, Donato B, Sparks TH, Pollard E (2000). Ecological correlates of island incidence and geographical range among British butterflies.. Biodiv Conserv.

[47] Pöyry J, Leinonen R, Söderman G, Nieminen M, Heikkinen RK (2011). Climate-induced increase of moth multivoltinism in boreal regions.. Global Ecol Biogeogr.

[48] Stearns SC (1983). The influence of size and phylogeny on patterns of covariation among life-history traits in the mammals.. Oikos.

[49] Bommarco R, Biesmeijer JC, Meyer B, Potts SG, Pöyry J (2010). Dispersal capacity and diet breadth modify the response of wild bees to habitat loss.. Proc R Soc B.

[50] Holt RD, Polis GA, Winemiller KO (1996). Food webs in space: an island biogeographic perspective.. Food webs: integration of patterns and dynamics.

[51] Beck J, Kitching IJ (2007). Correlates of range size and dispersal ability: a comparative analysis of sphingid moths from the Indo-Australian tropics.. Global Ecol Biogeogr.

[52] Powney GD, Roy DB, Chapman D, Oliver TH (2010). Synchrony of butterfly populations across species’ geographic ranges.. Oikos.

[53] Schweiger O, Heikkinen RK, Harpke A, Hickler T, Klotz S (2012). Increasing range mismatching of interacting species under global change is related to species traits.. Global Ecol Biogeogr.

[54] Parmesan C, Woiwod IP, Reynolds DR, Thomas CD (2001). Coping with modern times? Insect movement and climate change.. Insect movement: mechanisms and consequences.

[55] Sparks TH, Roy DB, Dennis RLH (2005). The influence of temperature on migration of Lepidoptera into Britain.. Global Change Biology.

[56] Nieminen M, Rita H, Uuvana P (1999). Body size and migration rate in moths.. Ecography.

[57] Loder N, Gaston KJ, Warren PH, Arnold HR (1998). Body size and feeding specificity: macrolepidoptera in Britain.. Biol J Linn Soc.

[58] Kreft H, Jetz W, Mutke J, Kier G, Barthlott W (2008). Global diversity of island floras from a macroecological perspective.. Ecol Letters.

[59] Chapman JW, Nesbit RL, Burgin LE, Reynolds DR, Smith AD (2010). Flight orientation behaviors promote optimal migration trajectories in high flying insects.. Science.

[60] Stadel Nielsen P (2008). Data registeret med automatiske lysfælder til natsommerfugle..

[61] Karsholt O, Razowski J (1996). The Lepidoptera of Europe - a distributional checklist..

[62] Nordström F (1943). Catalogus Insectorum Sueciae. III. Macrolepidoptera.. Opusc Ent.

[63] Svensson I, Elmquist H, Gustafsson B, Hellberg H, Imby L (1994). Catalogus Lepidopterorum Sueciae..

[64] Lindeborg M (2009). Intressanta fynd av storfjärilar (Macrolepidoptera) i Sverige 2008.. Ent Tidskr.

[65] Henriksen HJ, Kreutzer IB (1982). The butterflies of Scandinavia in nature..

[66] Palm E (1986). Nordeuropas Pyralider - med særligt henblick på den danske fauna (Lepidoptera: Pyralidae)..

[67] Emmet AM, Emmet AM, Heath J (1991). Life history and habits of the British Lepidoptera.. The moths and butterflies of Great Britain and Ireland.

[68] Huldén L, Albrecht A, Itämies J, Malinen P, Wettenhovi J (2000). Atlas of Finnish Macrolepidoptera..

[69] Hydén N (2007). Nationalnyckeln till Sveriges flora och fauna..

[70] Skou P (1984). Nordens Målare..

[71] Skou P (1991). Nordens ugler..

[72] Svensson I (1993). Lepidoptera-calender..

[73] Alexandersson H (2002). Temperatur och nederbörd i Sverige 1860–2001..

[74] Mutanen M, Wahlberg N, Kaila L (2010). Comprehensive gene and taxon coverage elucidates radiation patterns in moths and butterflies.. Proc R Soc B.

[75] Gelman A, Hill J (2007). Data analysis using regression and multilevel/hierarchical models..

[76] Bolker BM, Brooks ME, Clark CJ, Geange SW, Poulsen JR (2009). Generalized linear mixed models: a practical guide for ecology and evolution.. Trends Ecol Evol.

[77] Altermatt F (2010). Tell me what you eat and I will tell you when you fly: diet can predict phenological changes in response to climate change.. Ecol Letters.

[78] Gaston KJ, Reavey D (1989). Patterns in the life histories and feeding strategies of British Macrolepidoptera.. Biol J Linn Soc.

[79] Karsholt O, Nielsen PS (1998). Revideret katalog over de danske sommerfugle..

[80] Karsholt O, Bygebjerg R, Meedom P, Kjeldgaard S (2008). Anholts sommerfugle (Lepidoptera).. Ent Meddr.

