# Supplementary material for: Species-Area Relationships Are Controlled by Species Traits
Source: PLoS One. 2012 May 21;7(5):e37359. doi: 10.1371/journal.pone.0037359 (PMC3357413; doi:10.1371/journal.pone.0037359)
Supplement: Table S1 — Scientific names, the number of the eight islands where the species has been recorded and their taxonomic group. The list is sorted systematically according to Karsholt and Razowski [61]. (DOCX) [file pone.0037359.s001.docx]

Table S1.

| **Taxonomic group** | **Species** | **Number of islands where the species has been recorded** |
| --- | --- | --- |
| Others | *Triodia sylvina* | 5 |
| Others | *Korscheltellus lupulina* | 4 |
| Others | *Pharmacis fusconebulosa* | 5 |
| Others | *Phymatopus hecta* | 2 |
| Others | *Hepialus humuli* | 4 |
| Others | *Apoda limacodes* | 4 |
| Others | *Heterogenea asella* | 1 |
| Others | *Adscita statices* | 5 |
| Others | *Zygaena minos* | 1 |
| Others | *Zygaena viciae* | 1 |
| Others | *Zygaena filipendulae* | 5 |
| Others | *Zygaena lonicerae* | 5 |
| Others | *Cossus cossus* | 6 |
| Others | *Zeuzera pyrina* | 3 |
| Others | *Phragmataecia castaneae* | 2 |
| Pyralidae | *Aphomia sociella* | 6 |
| Pyralidae | *Aphomia zelleri* | 4 |
| Pyralidae | *Achroia grisella* | 4 |
| Pyralidae | *Galleria mellonella* | 5 |
| Pyralidae | *Synaphe punctalis* | 6 |
| Pyralidae | *Pyralis regalis* | 4 |
| Pyralidae | *Pyralis farinalis* | 6 |
| Pyralidae | *Aglossa pinguinalis* | 5 |
| Pyralidae | *Hypsopygia costalis* | 6 |
| Pyralidae | *Orthopygia glaucinalis* | 6 |
| Pyralidae | *Endotricha flammealis* | 7 |
| Pyralidae | *Cryptoblabes bistriga* | 6 |
| Pyralidae | *Elegia similella* | 4 |
| Pyralidae | *Ortholepis betulae* | 5 |
| Pyralidae | *Ortholepis vacciniella* | 1 |
| Pyralidae | *Pyla fusca* | 7 |
| Pyralidae | *Pempeliella ornatella* | 5 |
| Pyralidae | *Pempeliella dilutella* | 5 |
| Pyralidae | *Khorassania compositella* | 4 |
| Pyralidae | *Sciota hostilis* | 6 |
| Pyralidae | *Sciota adelphella* | 6 |
| Pyralidae | *Selagia spadicella* | 6 |
| Pyralidae | *Pima boisduvaliella* | 5 |
| Pyralidae | *Oncocera semirubella* | 4 |
| Pyralidae | *Oncocera faecella* | 5 |
| Pyralidae | *Pempelia formosa* | 7 |
| Pyralidae | *Pempelia palumbella* | 7 |
| Pyralidae | *Dioryctria sylvestrella* | 4 |
| Pyralidae | *Dioryctria schuetzeella* | 4 |
| Pyralidae | *Dioryctria simplicella* | 5 |
| Pyralidae | *Dioryctria abietella* | 8 |
| Pyralidae | *Phycita roborella* | 6 |
| Pyralidae | *Hypochalcia ahenella* | 6 |
| Pyralidae | *Nephopterix angustella* | 3 |
| Pyralidae | *Conobathra repandana* | 7 |
| Pyralidae | *Trachycera advenella* | 7 |
| Pyralidae | *Trachycera suavella* | 3 |
| Pyralidae | *Trachycera marmorea* | 4 |
| Pyralidae | *Acrobasis consociella* | 6 |
| Pyralidae | *Apomyelois bistriatella* | 3 |
| Pyralidae | *Apomyelois tetricella* | 3 |
| Pyralidae | *Apomyelois cirrigerella* | 3 |
| Pyralidae | *Myelois circumvoluta* | 7 |
| Pyralidae | *Zophodia grossulariella* | 4 |
| Pyralidae | *Assara terebrella* | 4 |
| Pyralidae | *Euzophera pinguis* | 5 |
| Pyralidae | *Euzophera cinerosella* | 6 |
| Pyralidae | *Euzophera fuliginosella* | 4 |
| Pyralidae | *Nyctegretis lineana* | 8 |
| Pyralidae | *Nyctegretis cinnamomella* | 2 |
| Pyralidae | *Homoeosoma nimbella* | 4 |
| Pyralidae | *Phycitodes maritima* | 3 |
| Pyralidae | *Phycitodes binaevella* | 6 |
| Pyralidae | *Phycitodes saxicola* | 5 |
| Pyralidae | *Phycitodes albatella* | 5 |
| Pyralidae | *Vitula biviella* | 5 |
| Pyralidae | *Ephestia elutella* | 6 |
| Pyralidae | *Anerastia lotella* | 7 |
| Pyralidae | *Scoparia subfusca* | 5 |
| Pyralidae | *Scoparia basistrigalis* | 3 |
| Pyralidae | *Scoparia ambigualis* | 6 |
| Pyralidae | *Scoparia ancipitella* | 3 |
| Pyralidae | *Scoparia conicella* | 1 |
| Pyralidae | *Scoparia pyralella* | 5 |
| Pyralidae | *Dipleurina lacustrata* | 6 |
| Pyralidae | *Eudonia murana* | 3 |
| Pyralidae | *Eudonia laetella* | 1 |
| Pyralidae | *Eudonia truncicolella* | 5 |
| Pyralidae | *Eudonia mercurella* | 5 |
| Pyralidae | *Eudonia sudetica* | 4 |
| Pyralidae | *Witlesia pallida* | 6 |
| Pyralidae | *Heliothela wulfeniana* | 1 |
| Pyralidae | *Chilo phragmitella* | 6 |
| Pyralidae | *Calamotropha paludella* | 7 |
| Pyralidae | *Chrysoteuchia culmella* | 8 |
| Pyralidae | *Crambus pascuella* | 7 |
| Pyralidae | *Crambus silvella* | 5 |
| Pyralidae | *Crambus scoticus* | 4 |
| Pyralidae | *Crambus ericella* | 4 |
| Pyralidae | *Crambus alienellus* | 1 |
| Pyralidae | *Crambus heringiellus* | 7 |
| Pyralidae | *Crambus pratella* | 3 |
| Pyralidae | *Crambus lathoniellus* | 7 |
| Pyralidae | *Crambus hamella* | 4 |
| Pyralidae | *Crambus perlella* | 6 |
| Pyralidae | *Agriphila deliella* | 4 |
| Pyralidae | *Agriphila tristella* | 8 |
| Pyralidae | *Agriphila inquinatella* | 7 |
| Pyralidae | *Agriphila latistria* | 1 |
| Pyralidae | *Agriphila selasella* | 5 |
| Pyralidae | *Agriphila straminella* | 8 |
| Pyralidae | *Agriphila poliellus* | 4 |
| Pyralidae | *Agriphila geniculea* | 4 |
| Pyralidae | *Catoptria permutatellus* | 5 |
| Pyralidae | *Catoptria osthelderi* | 4 |
| Pyralidae | *Catoptria pinella* | 8 |
| Pyralidae | *Catoptria margaritella* | 5 |
| Pyralidae | *Catoptria fulgidella* | 6 |
| Pyralidae | *Catoptria falsella* | 7 |
| Pyralidae | *Catoptria verellus* | 3 |
| Pyralidae | *Catoptria lythargyrella* | 3 |
| Pyralidae | *Thisanotia chrysonuchella* | 4 |
| Pyralidae | *Pediasia fascelinella* | 7 |
| Pyralidae | *Pediasia contaminella* | 6 |
| Pyralidae | *Pediasia aridella* | 8 |
| Pyralidae | *Platytes cerussella* | 5 |
| Pyralidae | *Platytes alpinella* | 7 |
| Pyralidae | *Schoenobius gigantella* | 6 |
| Pyralidae | *Donacaula forficella* | 2 |
| Pyralidae | *Donacaula mucronella* | 4 |
| Pyralidae | *Elophila nymphaeata* | 8 |
| Pyralidae | *Acentria ephemerella* | 3 |
| Pyralidae | *Cataclysta lemnata* | 5 |
| Pyralidae | *Parapoynx stratiotata* | 5 |
| Pyralidae | *Cynaeda dentalis* | 4 |
| Pyralidae | *Epascestria pustulalis* | 2 |
| Pyralidae | *Evergestis forficalis* | 6 |
| Pyralidae | *Evergestis extimalis* | 6 |
| Pyralidae | *Evergestis limbata* | 5 |
| Pyralidae | *Evergestis pallidata* | 6 |
| Pyralidae | *Evergestis aenealis* | 4 |
| Pyralidae | *Udea lutealis* | 8 |
| Pyralidae | *Udea prunalis* | 8 |
| Pyralidae | *Udea inquinatalis* | 2 |
| Pyralidae | *Udea olivalis* | 3 |
| Pyralidae | *Udea hamalis* | 3 |
| Pyralidae | *Opsibotys fuscalis* | 8 |
| Pyralidae | *Loxostege sticticalis* | 5 |
| Pyralidae | *Pyrausta cingulata* | 4 |
| Pyralidae | *Pyrausta sanguinalis* | 5 |
| Pyralidae | *Pyrausta despicata* | 7 |
| Pyralidae | *Pyrausta porphyralis* | 2 |
| Pyralidae | *Pyrausta aurata* | 7 |
| Pyralidae | *Pyrausta purpuralis* | 6 |
| Pyralidae | *Pyrausta ostrinalis* | 4 |
| Pyralidae | *Pyrausta nigrata* | 2 |
| Pyralidae | *Pyrausta aerealis* | 6 |
| Pyralidae | *Nascia cilialis* | 3 |
| Pyralidae | *Sitochroa palealis* | 6 |
| Pyralidae | *Sitochroa verticalis* | 5 |
| Pyralidae | *Perinephela lancealis* | 3 |
| Pyralidae | *Phlyctaenia coronata* | 6 |
| Pyralidae | *Phlyctaenia stachydalis* | 3 |
| Pyralidae | *Phlyctaenia perlucidalis* | 7 |
| Pyralidae | *Algedonia terrealis* | 5 |
| Pyralidae | *Psammotis pulveralis* | 7 |
| Pyralidae | *Ostrinia palustralis* | 3 |
| Pyralidae | *Ostrinia nubilalis* | 6 |
| Pyralidae | *Ebulea crocealis* | 3 |
| Pyralidae | *Anania verbascalis* | 4 |
| Pyralidae | *Anania funebris* | 1 |
| Pyralidae | *Eurrhypara hortulata* | 7 |
| Pyralidae | *Paratalanta pandalis* | 5 |
| Pyralidae | *Paratalanta hyalinalis* | 4 |
| Pyralidae | *Pleuroptya ruralis* | 8 |
| Pyralidae | *Mecyna flavalis* | 6 |
| Pyralidae | *Agrotera nemoralis* | 4 |
| Pyralidae | *Dolicharthria punctalis* | 5 |
| Pyralidae | *Nomophila noctuella* | 8 |
| Others | *Poecilocampa populi* | 6 |
| Others | *Trichiura crataegi* | 2 |
| Others | *Eriogaster lanestris* | 3 |
| Others | *Malacosoma neustria* | 7 |
| Others | *Malacosoma castrensis* | 7 |
| Others | *Lasiocampa trifolii* | 5 |
| Others | *Lasiocampa quercus* | 3 |
| Others | *Macrothylacia rubi* | 4 |
| Others | *Dendrolimus pini* | 7 |
| Others | *Euthrix potatoria* | 5 |
| Others | *Cosmotriche lobulina* | 1 |
| Others | *Phyllodesma ilicifolia* | 2 |
| Others | *Gastropacha quercifolia* | 3 |
| Others | *Endromis versicolora* | 4 |
| Others | *Aglia tau* | 1 |
| Others | *Saturnia pavonia* | 3 |
| Others | *Lemonia dumi* | 3 |
| Others | *Mimas tiliae* | 4 |
| Others | *Smerinthus ocellata* | 7 |
| Others | *Laothoe populi* | 7 |
| Others | *Sphinx ligustri* | 6 |
| Others | *Hyloicus pinastri* | 7 |
| Others | *Hemaris tityus* | 4 |
| Others | *Hemaris fuciformis* | 5 |
| Others | *Hyles galii* | 7 |
| Others | *Deilephila elpenor* | 7 |
| Others | *Deilephila porcellus* | 8 |
| Butterflies | *Erynnis tages* | 3 |
| Butterflies | *Pyrgus malvae* | 3 |
| Butterflies | *Pyrgus armoricanus* | 1 |
| Butterflies | *Pyrgus alveus* | 3 |
| Butterflies | *Carterocephalus silvicola* | 2 |
| Butterflies | *Thymelicus lineola* | 4 |
| Butterflies | *Hesperia comma* | 4 |
| Butterflies | *Ochlodes venata* | 2 |
| Butterflies | *Parnassius mnemosyne* | 1 |
| Butterflies | *Parnassius apollo* | 2 |
| Butterflies | *Papilio machaon* | 6 |
| Butterflies | *Leptidea sinapis* | 3 |
| Butterflies | *Leptidea reali* | 3 |
| Butterflies | *Anthocharis cardamines* | 5 |
| Butterflies | *Aporia crataegi* | 4 |
| Butterflies | *Pieris brassicae* | 7 |
| Butterflies | *Pieris rapae* | 8 |
| Butterflies | *Pieris napi* | 7 |
| Butterflies | *Pontia daplidice* | 3 |
| Butterflies | *Colias palaeno* | 4 |
| Butterflies | *Gonepteryx rhamni* | 6 |
| Butterflies | *Hamearis lucina* | 1 |
| Butterflies | *Lycaena phlaeas* | 7 |
| Butterflies | *Lycaena virgaureae* | 5 |
| Butterflies | *Lycaena hippothoe* | 3 |
| Butterflies | *Thecla betulae* | 5 |
| Butterflies | *Neozephyrus quercus* | 4 |
| Butterflies | *Callophrys rubi* | 4 |
| Butterflies | *Satyrium w-album* | 5 |
| Butterflies | *Satyrium pruni* | 1 |
| Butterflies | *Cupido minimus* | 5 |
| Butterflies | *Celastrina argiolus* | 6 |
| Butterflies | *Glaucopsyche alexis* | 1 |
| Butterflies | *Maculinea arion* | 3 |
| Butterflies | *Plebeius argus* | 5 |
| Butterflies | *Plebeius idas* | 5 |
| Butterflies | *Plebeius argyrognomon* | 1 |
| Butterflies | *Vacciniina optilete* | 3 |
| Butterflies | *Aricia eumedon* | 2 |
| Butterflies | *Aricia agestis* | 3 |
| Butterflies | *Aricia artaxerxes* | 4 |
| Butterflies | *Cyaniris semiargus* | 4 |
| Butterflies | *Polyommatus dorylas* | 2 |
| Butterflies | *Polyommatus amandus* | 5 |
| Butterflies | *Polyommatus icarus* | 6 |
| Butterflies | *Argynnis paphia* | 5 |
| Butterflies | *Argynnis aglaja* | 5 |
| Butterflies | *Argynnis adippe* | 6 |
| Butterflies | *Argynnis niobe* | 5 |
| Butterflies | *Issoria lathonia* | 4 |
| Butterflies | *Brenthis ino* | 3 |
| Butterflies | *Clossiana euphrosyne* | 2 |
| Butterflies | *Clossiana selene* | 4 |
| Butterflies | *Boloria aquilonaris* | 2 |
| Butterflies | *Inachis io* | 7 |
| Butterflies | *Aglais urticae* | 7 |
| Butterflies | *Polygonia c-album* | 6 |
| Butterflies | *Araschnia levana* | 3 |
| Butterflies | *Nymphalis antiopa* | 8 |
| Butterflies | *Nymphalis polychloros* | 5 |
| Butterflies | *Euphydryas aurinia* | 2 |
| Butterflies | *Melitaea cinxia* | 4 |
| Butterflies | *Melitaea diamina* | 2 |
| Butterflies | *Melitaea britomartis* | 1 |
| Butterflies | *Melitaea athalia* | 2 |
| Butterflies | *Limenitis populi* | 4 |
| Butterflies | *Limenitis camilla* | 1 |
| Butterflies | *Apatura iris* | 2 |
| Butterflies | *Pararge aegeria* | 5 |
| Butterflies | *Lasiommata megera* | 5 |
| Butterflies | *Lasiommata petropolitana* | 1 |
| Butterflies | *Lasiommata maera* | 2 |
| Butterflies | *Lopinga achine* | 1 |
| Butterflies | *Coenonympha tullia* | 2 |
| Butterflies | *Coenonympha arcania* | 2 |
| Butterflies | *Coenonympha pamphilus* | 6 |
| Butterflies | *Aphantopus hyperantus* | 6 |
| Butterflies | *Maniola jurtina* | 6 |
| Butterflies | *Erebia ligea* | 2 |
| Butterflies | *Hipparchia semele* | 6 |
| Geometridae | *Thyatira batis* | 7 |
| Geometridae | *Habrosyne pyritoides* | 6 |
| Geometridae | *Tethea ocularis* | 2 |
| Geometridae | *Tethea or* | 7 |
| Geometridae | *Tetheella fluctuosa* | 7 |
| Geometridae | *Ochropacha duplaris* | 7 |
| Geometridae | *Cymatophorina diluta* | 2 |
| Geometridae | *Polyploca ridens* | 1 |
| Geometridae | *Achyla flavicornis* | 5 |
| Geometridae | *Falcaria lacertinaria* | 6 |
| Geometridae | *Watsonalla binaria* | 7 |
| Geometridae | *Watsonalla cultraria* | 3 |
| Geometridae | *Drepana curvatula* | 7 |
| Geometridae | *Drepana falcataria* | 7 |
| Geometridae | *Sabra harpagula* | 2 |
| Geometridae | *Cilix glaucata* | 6 |
| Geometridae | *Archiearis parthenias* | 4 |
| Geometridae | *Archiearis notha* | 2 |
| Geometridae | *Abraxas grossulariata* | 8 |
| Geometridae | *Calospilos sylvata* | 6 |
| Geometridae | *Lomaspilis marginata* | 6 |
| Geometridae | *Ligdia adustata* | 5 |
| Geometridae | *Macaria notata* | 6 |
| Geometridae | *Macaria alternata* | 6 |
| Geometridae | *Macaria signaria* | 5 |
| Geometridae | *Macaria liturata* | 7 |
| Geometridae | *Macaria wauaria* | 6 |
| Geometridae | *Macaria carbonaria* | 1 |
| Geometridae | *Chiasmia clathrata* | 8 |
| Geometridae | *Itame brunneata* | 6 |
| Geometridae | *Cepphis advenaria* | 5 |
| Geometridae | *Petrophora chlorosata* | 5 |
| Geometridae | *Plagodis pulveraria* | 4 |
| Geometridae | *Plagodis dolabraria* | 7 |
| Geometridae | *Pachycnemia hippocastanaria* | 2 |
| Geometridae | *Opisthograptis luteolata* | 7 |
| Geometridae | *Epione repandaria* | 7 |
| Geometridae | *Epione vespertaria* | 7 |
| Geometridae | *Pseudopanthera macularia* | 1 |
| Geometridae | *Apeira syringaria* | 6 |
| Geometridae | *Ennomos autumnaria* | 4 |
| Geometridae | *Ennomos quercinaria* | 3 |
| Geometridae | *Ennomos alniaria* | 7 |
| Geometridae | *Ennomos fuscantaria* | 6 |
| Geometridae | *Ennomos erosaria* | 6 |
| Geometridae | *Selenia dentaria* | 6 |
| Geometridae | *Selenia lunularia* | 4 |
| Geometridae | *Selenia tetralunaria* | 6 |
| Geometridae | *Odontopera bidentata* | 7 |
| Geometridae | *Crocallis elinguaria* | 8 |
| Geometridae | *Ourapteryx sambucaria* | 5 |
| Geometridae | *Colotois pennaria* | 5 |
| Geometridae | *Angerona prunaria* | 5 |
| Geometridae | *Apocheima hispidaria* | 2 |
| Geometridae | *Apocheima pilosaria* | 5 |
| Geometridae | *Lycia hirtaria* | 5 |
| Geometridae | *Biston strataria* | 5 |
| Geometridae | *Biston betularia* | 7 |
| Geometridae | *Agriopis leucophaearia* | 4 |
| Geometridae | *Agriopis aurantiaria* | 5 |
| Geometridae | *Agriopis marginaria* | 5 |
| Geometridae | *Erannis defoliaria* | 7 |
| Geometridae | *Peribatodes rhomboidaria* | 7 |
| Geometridae | *Peribatodes secundaria* | 7 |
| Geometridae | *Selidosema brunnearia* | 5 |
| Geometridae | *Cleora cinctaria* | 6 |
| Geometridae | *Deileptenia ribeata* | 4 |
| Geometridae | *Alcis repandata* | 7 |
| Geometridae | *Alcis jubata* | 4 |
| Geometridae | *Arichanna melanaria* | 6 |
| Geometridae | *Hypomecis roboraria* | 6 |
| Geometridae | *Hypomecis punctinalis* | 4 |
| Geometridae | *Cleorodes lichenaria* | 7 |
| Geometridae | *Fagivorina arenaria* | 2 |
| Geometridae | *Ectropis crepuscularia* | 7 |
| Geometridae | *Paradarisa consonaria* | 2 |
| Geometridae | *Parectropis similaria* | 2 |
| Geometridae | *Aethalura punctulata* | 5 |
| Geometridae | *Ematurga atomaria* | 6 |
| Geometridae | *Bupalus piniaria* | 7 |
| Geometridae | *Cabera pusaria* | 7 |
| Geometridae | *Cabera exanthemata* | 8 |
| Geometridae | *Lomographa bimaculata* | 5 |
| Geometridae | *Lomographa temerata* | 6 |
| Geometridae | *Theria rupicapraria* | 1 |
| Geometridae | *Campaea margaritata* | 7 |
| Geometridae | *Hylaea fasciaria* | 6 |
| Geometridae | *Gnophos obfuscata* | 2 |
| Geometridae | *Charissa obscurata* | 6 |
| Geometridae | *Parietaria vittaria* | 1 |
| Geometridae | *Siona lineata* | 6 |
| Geometridae | *Dyscia fagaria* | 4 |
| Geometridae | *Perconia strigillaria* | 4 |
| Geometridae | *Epirranthis diversata* | 2 |
| Geometridae | *Alsophila aescularia* | 4 |
| Geometridae | *Pseudoterpna pruinata* | 1 |
| Geometridae | *Geometra papilionaria* | 6 |
| Geometridae | *Comibaena bajularia* | 6 |
| Geometridae | *Hemithea aestivaria* | 6 |
| Geometridae | *Chlorissa viridata* | 5 |
| Geometridae | *Thalera fimbrialis* | 5 |
| Geometridae | *Hemistola chrysoprasaria* | 3 |
| Geometridae | *Jodis lactearia* | 5 |
| Geometridae | *Jodis putata* | 4 |
| Geometridae | *Cyclophora pendularia* | 5 |
| Geometridae | *Cyclophora albipunctata* | 7 |
| Geometridae | *Cyclophora porata* | 3 |
| Geometridae | *Cyclophora quercimontaria* | 5 |
| Geometridae | *Cyclophora punctaria* | 8 |
| Geometridae | *Cyclophora linearia* | 5 |
| Geometridae | *Timandra griseata* | 6 |
| Geometridae | *Timandra comae* | 7 |
| Geometridae | *Scopula immorata* | 4 |
| Geometridae | *Scopula nigropunctata* | 1 |
| Geometridae | *Scopula virgulata* | 2 |
| Geometridae | *Scopula ornata* | 4 |
| Geometridae | *Scopula decorata* | 4 |
| Geometridae | *Scopula rubiginata* | 7 |
| Geometridae | *Scopula marginepunctata* | 1 |
| Geometridae | *Scopula incanata* | 4 |
| Geometridae | *Scopula immutata* | 8 |
| Geometridae | *Scopula ternata* | 4 |
| Geometridae | *Scopula floslactata* | 5 |
| Geometridae | *Idaea ochrata* | 4 |
| Geometridae | *Idaea serpentata* | 4 |
| Geometridae | *Idaea muricata* | 5 |
| Geometridae | *Idaea sylvestraria* | 6 |
| Geometridae | *Idaea biselata* | 6 |
| Geometridae | *Idaea fuscovenosa* | 4 |
| Geometridae | *Idaea humiliata* | 4 |
| Geometridae | *Idaea seriata* | 6 |
| Geometridae | *Idaea dimidiata* | 7 |
| Geometridae | *Idaea pallidata* | 4 |
| Geometridae | *Idaea trigeminata* | 1 |
| Geometridae | *Idaea emarginata* | 5 |
| Geometridae | *Idaea aversata* | 7 |
| Geometridae | *Idaea straminata* | 6 |
| Geometridae | *Idaea deversaria* | 5 |
| Geometridae | *Rhodostrophia vibicaria* | 7 |
| Geometridae | *Lythria cruentaria* | 5 |
| Geometridae | *Phibalapteryx virgata* | 7 |
| Geometridae | *Scotopteryx chenopodiata* | 6 |
| Geometridae | *Scotopteryx mucronata* | 1 |
| Geometridae | *Scotopteryx luridata* | 1 |
| Geometridae | *Orthonama vittata* | 7 |
| Geometridae | *Xanthorhoe biriviata* | 4 |
| Geometridae | *Xanthorhoe designata* | 5 |
| Geometridae | *Xanthorhoe decoloraria* | 1 |
| Geometridae | *Xanthorhoe spadicearia* | 7 |
| Geometridae | *Xanthorhoe ferrugata* | 8 |
| Geometridae | *Xanthorhoe quadrifasciata* | 8 |
| Geometridae | *Xanthorhoe montanata* | 8 |
| Geometridae | *Xanthorhoe fluctuata* | 8 |
| Geometridae | *Catarhoe rubidata* | 3 |
| Geometridae | *Catarhoe cuculata* | 7 |
| Geometridae | *Epirrhoe hastulata* | 2 |
| Geometridae | *Epirrhoe pupillata* | 1 |
| Geometridae | *Epirrhoe tristata* | 6 |
| Geometridae | *Epirrhoe alternata* | 8 |
| Geometridae | *Epirrhoe rivata* | 6 |
| Geometridae | *Anticlea derivata* | 5 |
| Geometridae | *Epirrhoe galiata* | 6 |
| Geometridae | *Costaconvexa polygrammata* | 4 |
| Geometridae | *Camptogramma bilineata* | 7 |
| Geometridae | *Entephria caesiata* | 2 |
| Geometridae | *Larentia clavaria* | 6 |
| Geometridae | *Anticlea badiata* | 6 |
| Geometridae | *Mesoleuca albicillata* | 6 |
| Geometridae | *Pelurga comitata* | 8 |
| Geometridae | *Lampropteryx suffumata* | 5 |
| Geometridae | *Cosmorhoe ocellata* | 8 |
| Geometridae | *Eulithis prunata* | 6 |
| Geometridae | *Eulithis testata* | 7 |
| Geometridae | *Eulithis populata* | 5 |
| Geometridae | *Eulithis mellinata* | 6 |
| Geometridae | *Eulithis pyraliata* | 5 |
| Geometridae | *Ecliptopera silaceata* | 7 |
| Geometridae | *Ecliptopera capitata* | 3 |
| Geometridae | *Chloroclysta siterata* | 7 |
| Geometridae | *Chloroclysta miata* | 6 |
| Geometridae | *Chloroclysta citrata* | 7 |
| Geometridae | *Chloroclysta latefasciata* | 5 |
| Geometridae | *Chloroclysta truncata* | 7 |
| Geometridae | *Cidaria fulvata* | 7 |
| Geometridae | *Plemyria rubiginata* | 7 |
| Geometridae | *Pennithera firmata* | 6 |
| Geometridae | *Thera obeliscata* | 7 |
| Geometridae | *Thera variata* | 7 |
| Geometridae | *Thera britannica* | 3 |
| Geometridae | *Thera cognata* | 8 |
| Geometridae | *Thera juniperata* | 6 |
| Geometridae | *Thera serraria* | 1 |
| Geometridae | *Eustroma reticulata* | 4 |
| Geometridae | *Electrophaes corylata* | 6 |
| Geometridae | *Colostygia olivata* | 3 |
| Geometridae | *Colostygia pectinataria* | 7 |
| Geometridae | *Hydriomena furcata* | 7 |
| Geometridae | *Hydriomena impluviata* | 5 |
| Geometridae | *Hydriomena ruberata* | 5 |
| Geometridae | *Coenocalpe lapidata* | 1 |
| Geometridae | *Horisme vitalbata* | 4 |
| Geometridae | *Horisme tersata* | 5 |
| Geometridae | *Horisme aemulata* | 2 |
| Geometridae | *Horisme aquata* | 1 |
| Geometridae | *Melanthia procellata* | 1 |
| Geometridae | *Pareulype berberata* | 3 |
| Geometridae | *Spargania luctuata* | 5 |
| Geometridae | *Rheumaptera hastata* | 5 |
| Geometridae | *Rheumaptera subhastata* | 2 |
| Geometridae | *Rheumaptera cervinalis* | 3 |
| Geometridae | *Rheumaptera undulata* | 7 |
| Geometridae | *Triphosa dubitata* | 7 |
| Geometridae | *Philereme vetulata* | 4 |
| Geometridae | *Philereme transversata* | 4 |
| Geometridae | *Euphyia biangulata* | 4 |
| Geometridae | *Euphyia unangulata* | 7 |
| Geometridae | *Epirrita dilutata* | 7 |
| Geometridae | *Epirrita christyi* | 5 |
| Geometridae | *Epirrita autumnata* | 6 |
| Geometridae | *Operophtera brumata* | 4 |
| Geometridae | *Operophtera fagata* | 4 |
| Geometridae | *Perizoma taeniata* | 2 |
| Geometridae | *Perizoma affinitata* | 2 |
| Geometridae | *Perizoma alchemillata* | 8 |
| Geometridae | *Perizoma hydrata* | 4 |
| Geometridae | *Perizoma bifaciata* | 6 |
| Geometridae | *Perizoma minorata* | 1 |
| Geometridae | *Perizoma blandiata* | 7 |
| Geometridae | *Perizoma albulata* | 5 |
| Geometridae | *Perizoma flavofasciata* | 7 |
| Geometridae | *Perizoma didymata* | 5 |
| Geometridae | *Perizoma sagittata* | 5 |
| Geometridae | *Perizoma parallelolineata* | 4 |
| Geometridae | *Eupithecia tenuiata* | 6 |
| Geometridae | *Eupithecia inturbata* | 5 |
| Geometridae | *Eupithecia haworthiata* | 4 |
| Geometridae | *Eupithecia immundata* | 2 |
| Geometridae | *Eupithecia plumbeolata* | 4 |
| Geometridae | *Eupithecia abietaria* | 7 |
| Geometridae | *Eupithecia analoga* | 4 |
| Geometridae | *Eupithecia linariata* | 7 |
| Geometridae | *Eupithecia irriguata* | 4 |
| Geometridae | *Eupithecia exiguata* | 7 |
| Geometridae | *Eupithecia insigniata* | 3 |
| Geometridae | *Eupithecia valerianata* | 4 |
| Geometridae | *Eupithecia pygmaeata* | 4 |
| Geometridae | *Eupithecia venosata* | 5 |
| Geometridae | *Eupithecia egenaria* | 4 |
| Geometridae | *Eupithecia centaureata* | 8 |
| Geometridae | *Eupithecia actaeata* | 2 |
| Geometridae | *Eupithecia selinata* | 2 |
| Geometridae | *Eupithecia trisignaria* | 7 |
| Geometridae | *Eupithecia intricata* | 6 |
| Geometridae | *Eupithecia cauchiata* | 1 |
| Geometridae | *Eupithecia satyrata* | 6 |
| Geometridae | *Eupithecia absinthiata* | 7 |
| Geometridae | *Eupithecia goossensiata* | 2 |
| Geometridae | *Eupithecia assimilata* | 6 |
| Geometridae | *Eupithecia vulgata* | 7 |
| Geometridae | *Eupithecia tripunctaria* | 6 |
| Geometridae | *Eupithecia denotata* | 4 |
| Geometridae | *Eupithecia subfuscata* | 7 |
| Geometridae | *Eupithecia icterata* | 7 |
| Geometridae | *Eupithecia succenturiata* | 8 |
| Geometridae | *Eupithecia orphnata* | 3 |
| Geometridae | *Eupithecia subumbrata* | 7 |
| Geometridae | *Eupithecia millefoliata* | 4 |
| Geometridae | *Eupithecia simpliciata* | 5 |
| Geometridae | *Eupithecia distinctaria* | 3 |
| Geometridae | *Eupithecia sinuosaria* | 6 |
| Geometridae | *Eupithecia indigata* | 6 |
| Geometridae | *Eupithecia pimpinellata* | 5 |
| Geometridae | *Eupithecia nanata* | 6 |
| Geometridae | *Eupithecia innotata* | 6 |
| Geometridae | *Eupithecia ochridata* | 4 |
| Geometridae | *Eupithecia virgaureata* | 3 |
| Geometridae | *Eupithecia abbreviata* | 4 |
| Geometridae | *Eupithecia dodoneata* | 5 |
| Geometridae | *Eupithecia pusillata* | 8 |
| Geometridae | *Eupithecia lanceata* | 3 |
| Geometridae | *Eupithecia lariciata* | 6 |
| Geometridae | *Eupithecia tantillaria* | 6 |
| Geometridae | *Eupithecia conterminata* | 2 |
| Geometridae | *Gymnoscelis rufifasciata* | 6 |
| Geometridae | *Chloroclystis v-ata* | 6 |
| Geometridae | *Rhinoprora rectangulata* | 5 |
| Geometridae | *Rhinoprora chloerata* | 4 |
| Geometridae | *Rhinoprora debiliata* | 3 |
| Geometridae | *Anticollix sparsata* | 4 |
| Geometridae | *Chesias legatella* | 6 |
| Geometridae | *Chesias rufata* | 1 |
| Geometridae | *Carsia sororiata* | 4 |
| Geometridae | *Aplocera plagiata* | 8 |
| Geometridae | *Aplocera efformata* | 4 |
| Geometridae | *Odezia atrata* | 4 |
| Geometridae | *Lithostege griseata* | 2 |
| Geometridae | *Lithostege farinata* | 2 |
| Geometridae | *Discoloxia blomeri* | 2 |
| Geometridae | *Venusia cambrica* | 5 |
| Geometridae | *Euchoeca nebulata* | 6 |
| Geometridae | *Asthena albulata* | 6 |
| Geometridae | *Hydrelia flammeolaria* | 6 |
| Geometridae | *Hydrelia sylvata* | 1 |
| Geometridae | *Lobophora halterata* | 6 |
| Geometridae | *Trichopteryx polycommata* | 1 |
| Geometridae | *Trichopteryx carpinata* | 5 |
| Geometridae | *Pterapherapteryx sexalata* | 6 |
| Geometridae | *Nothocasis sertata* | 1 |
| Geometridae | *Acasis viretata* | 6 |
| Others | *Thaumetopoea pinivora* | 3 |
| Others | *Pygaera timon* | 1 |
| Others | *Clostera curtula* | 7 |
| Others | *Clostera pigra* | 6 |
| Others | *Clostera anachoreta* | 1 |
| Others | *Clostera anastomosis* | 4 |
| Others | *Cerura vinula* | 5 |
| Others | *Furcula furcula* | 4 |
| Others | *Furcula bicuspis* | 4 |
| Others | *Furcula bifida* | 4 |
| Others | *Notodonta dromedarius* | 7 |
| Others | *Notodonta torva* | 3 |
| Others | *Notodonta tritophus* | 5 |
| Others | *Notodonta ziczac* | 7 |
| Others | *Drymonia dodonaea* | 2 |
| Others | *Drymonia ruficornis* | 2 |
| Others | *Pheosia tremula* | 6 |
| Others | *Pheosia gnoma* | 7 |
| Others | *Pterostoma palpina* | 7 |
| Others | *Ptilophora plumigera* | 1 |
| Others | *Leucodonta bicoloria* | 3 |
| Others | *Ptilodon capucina* | 7 |
| Others | *Odontosia carmelita* | 3 |
| Others | *Gluphisia crenata* | 4 |
| Others | *Phalera bucephala* | 8 |
| Others | *Peridea anceps* | 5 |
| Others | *Stauropus fagi* | 6 |
| Others | *Harpyia milhauseri* | 2 |
| Noctuidae | *Moma alpium* | 6 |
| Noctuidae | *Acronicta alni* | 6 |
| Noctuidae | *Acronicta cuspis* | 3 |
| Noctuidae | *Acronicta tridens* | 4 |
| Noctuidae | *Acronicta psi* | 7 |
| Noctuidae | *Acronicta aceris* | 6 |
| Noctuidae | *Acronicta leporina* | 7 |
| Noctuidae | *Acronicta megacephala* | 7 |
| Noctuidae | *Acronicta strigosa* | 4 |
| Noctuidae | *Acronicta menyanthidis* | 5 |
| Noctuidae | *Acronicta auricoma* | 7 |
| Noctuidae | *Acronicta euphorbiae* | 8 |
| Noctuidae | *Acronicta rumicis* | 7 |
| Noctuidae | *Craniophora ligustri* | 5 |
| Noctuidae | *Simyra albovenosa* | 6 |
| Noctuidae | *Cryphia raptricula* | 6 |
| Noctuidae | *Cryphia domestica* | 4 |
| Noctuidae | *Paracolax tristalis* | 5 |
| Noctuidae | *Macrochilo cribrumalis* | 6 |
| Noctuidae | *Herminia grisealis* | 6 |
| Noctuidae | *Polypogon tentacularia* | 3 |
| Noctuidae | *Pechipogo strigilata* | 5 |
| Noctuidae | *Zanclognatha lunalis* | 2 |
| Noctuidae | *Zanclognatha tarsipennalis* | 7 |
| Noctuidae | *Hypenodes humidalis* | 5 |
| Noctuidae | *Schrankia costaestrigalis* | 6 |
| Noctuidae | *Schrankia taenialis* | 2 |
| Noctuidae | *Catocala sponsa* | 7 |
| Noctuidae | *Catocala fraxini* | 7 |
| Noctuidae | *Catocala nupta* | 8 |
| Noctuidae | *Catocala promissa* | 7 |
| Noctuidae | *Catocala pacta* | 3 |
| Noctuidae | *Minucia lunaris* | 2 |
| Noctuidae | *Lygephila pastinum* | 5 |
| Noctuidae | *Lygephila viciae* | 4 |
| Noctuidae | *Lygephila craccae* | 7 |
| Noctuidae | *Tyta luctuosa* | 4 |
| Noctuidae | *Callistege mi* | 6 |
| Noctuidae | *Euclidia glyphica* | 4 |
| Noctuidae | *Laspeyria flexula* | 6 |
| Noctuidae | *Scoliopteryx libatrix* | 8 |
| Noctuidae | *Hypena proboscidalis* | 6 |
| Noctuidae | *Hypena rostralis* | 4 |
| Noctuidae | *Hypena crassalis* | 5 |
| Noctuidae | *Phytometra viridaria* | 3 |
| Noctuidae | *Rivula sericealis* | 8 |
| Noctuidae | *Parascotia fuliginaria* | 7 |
| Noctuidae | *Colobochyla salicalis* | 4 |
| Noctuidae | *Polychrysia moneta* | 4 |
| Noctuidae | *Lamprotes c-aureum* | 2 |
| Noctuidae | *Diachrysia chrysitis* | 8 |
| Noctuidae | *Diachrysia tutti* | 2 |
| Noctuidae | *Plusia festucae* | 7 |
| Noctuidae | *Plusia putnami* | 6 |
| Noctuidae | *Autographa macrogamma* | 2 |
| Noctuidae | *Autographa pulchrina* | 7 |
| Noctuidae | *Autographa buraetica* | 6 |
| Noctuidae | *Autographa jota* | 5 |
| Noctuidae | *Autographa bractea* | 6 |
| Noctuidae | *Syngrapha microgamma* | 1 |
| Noctuidae | *Syngrapha interrogationis* | 8 |
| Noctuidae | *Abrostola tripartita* | 8 |
| Noctuidae | *Abrostola asclepiadis* | 4 |
| Noctuidae | *Emmelia trabealis* | 4 |
| Noctuidae | *Protodeltote pygarga* | 8 |
| Noctuidae | *Deltote deceptoria* | 4 |
| Noctuidae | *Deltote uncula* | 6 |
| Noctuidae | *Deltote bankiana* | 8 |
| Noctuidae | *Eublemma minutata* | 5 |
| Noctuidae | *Trisateles emortualis* | 4 |
| Noctuidae | *Cucullia fraudatrix* | 3 |
| Noctuidae | *Cucullia absinthii* | 7 |
| Noctuidae | *Cucullia argentea* | 3 |
| Noctuidae | *Cucullia artemisiae* | 4 |
| Noctuidae | *Cucullia lactucae* | 3 |
| Noctuidae | *Cucullia lucifuga* | 2 |
| Noctuidae | *Cucullia umbratica* | 7 |
| Noctuidae | *Cucullia chamomillae* | 4 |
| Noctuidae | *Cucullia gnaphalii* | 1 |
| Noctuidae | *Cucullia asteris* | 5 |
| Noctuidae | *Shargacucullia scrophulariae* | 3 |
| Noctuidae | *Shargacucullia lychnitis* | 2 |
| Noctuidae | *Shargacucullia verbasci* | 2 |
| Noctuidae | *Calophasia lunula* | 5 |
| Noctuidae | *Amphipyra pyramidea* | 7 |
| Noctuidae | *Amphipyra berbera* | 6 |
| Noctuidae | *Amphipyra perflua* | 3 |
| Noctuidae | *Amphipyra tragopoginis* | 8 |
| Noctuidae | *Asteroscopus sphinx* | 4 |
| Noctuidae | *Brachionycha nubeculosa* | 1 |
| Noctuidae | *Diloba caeruleocephala* | 6 |
| Noctuidae | *Panemeria tenebrata* | 1 |
| Noctuidae | *Heliothis viriplaca* | 5 |
| Noctuidae | *Pyrrhia umbra* | 8 |
| Noctuidae | *Elaphria venustula* | 6 |
| Noctuidae | *Caradrina morpheus* | 7 |
| Noctuidae | *Platyperigea montana* | 6 |
| Noctuidae | *Paradrina selini* | 7 |
| Noctuidae | *Paradrina clavipalpis* | 5 |
| Noctuidae | *Hoplodrina octogenaria* | 7 |
| Noctuidae | *Hoplodrina blanda* | 7 |
| Noctuidae | *Hoplodrina ambigua* | 4 |
| Noctuidae | *Charanyca trigrammica* | 7 |
| Noctuidae | *Chilodes maritima* | 6 |
| Noctuidae | *Athetis gluteosa* | 2 |
| Noctuidae | *Athetis pallustris* | 5 |
| Noctuidae | *Proxenus lepigone* | 5 |
| Noctuidae | *Dypterygia scabriuscula* | 7 |
| Noctuidae | *Rusina ferruginea* | 7 |
| Noctuidae | *Thalpophila matura* | 8 |
| Noctuidae | *Trachea atriplicis* | 8 |
| Noctuidae | *Euplexia lucipara* | 7 |
| Noctuidae | *Phlogophora meticulosa* | 8 |
| Noctuidae | *Hyppa rectilinea* | 5 |
| Noctuidae | *Actinotia polyodon* | 7 |
| Noctuidae | *Chloantha hyperici* | 2 |
| Noctuidae | *Ipimorpha retusa* | 5 |
| Noctuidae | *Ipimorpha subtusa* | 6 |
| Noctuidae | *Enargia paleacea* | 7 |
| Noctuidae | *Parastichtis suspecta* | 8 |
| Noctuidae | *Parastichtis ypsillon* | 6 |
| Noctuidae | *Mesogona oxalina* | 3 |
| Noctuidae | *Dicycla oo* | 1 |
| Noctuidae | *Cosmia diffinis* | 1 |
| Noctuidae | *Cosmia affinis* | 4 |
| Noctuidae | *Cosmia pyralina* | 6 |
| Noctuidae | *Cosmia trapezina* | 8 |
| Noctuidae | *Xanthia togata* | 7 |
| Noctuidae | *Xanthia aurago* | 6 |
| Noctuidae | *Xanthia icteritia* | 7 |
| Noctuidae | *Xanthia gilvago* | 7 |
| Noctuidae | *Xanthia ocellaris* | 3 |
| Noctuidae | *Xanthia citrago* | 3 |
| Noctuidae | *Agrochola lychnidis* | 4 |
| Noctuidae | *Agrochola circellaris* | 8 |
| Noctuidae | *Agrochola lota* | 6 |
| Noctuidae | *Agrochola macilenta* | 6 |
| Noctuidae | *Agrochola nitida* | 5 |
| Noctuidae | *Agrochola helvola* | 7 |
| Noctuidae | *Agrochola litura* | 5 |
| Noctuidae | *Eupsilia transversa* | 6 |
| Noctuidae | *Conistra vaccinii* | 6 |
| Noctuidae | *Conistra rubiginosa* | 6 |
| Noctuidae | *Conistra rubiginea* | 6 |
| Noctuidae | *Conistra erythrocephala* | 6 |
| Noctuidae | *Dasypolia templi* | 6 |
| Noctuidae | *Brachylomia viminalis* | 7 |
| Noctuidae | *Aporophyla lutulenta* | 8 |
| Noctuidae | *Lithomoia solidaginis* | 6 |
| Noctuidae | *Lithophane socia* | 5 |
| Noctuidae | *Lithophane ornitopus* | 5 |
| Noctuidae | *Lithophane furcifera* | 5 |
| Noctuidae | *Lithophane lamda* | 4 |
| Noctuidae | *Lithophane consocia* | 4 |
| Noctuidae | *Xylena vetusta* | 6 |
| Noctuidae | *Xylena exsoleta* | 7 |
| Noctuidae | *Xylocampa areola* | 2 |
| Noctuidae | *Allophyes oxyacanthae* | 7 |
| Noctuidae | *Dichonia aprilina* | 7 |
| Noctuidae | *Dryobotodes eremita* | 8 |
| Noctuidae | *Antitype chi* | 7 |
| Noctuidae | *Ammoconia caecimacula* | 8 |
| Noctuidae | *Polymixis polymita* | 6 |
| Noctuidae | *Polymixis flavicincta* | 1 |
| Noctuidae | *Polymixis gemmea* | 6 |
| Noctuidae | *Blepharita satura* | 7 |
| Noctuidae | *Mniotype adusta* | 6 |
| Noctuidae | *Apamea monoglypha* | 8 |
| Noctuidae | *Apamea lithoxylaea* | 8 |
| Noctuidae | *Apamea sublustris* | 5 |
| Noctuidae | *Apamea crenata* | 8 |
| Noctuidae | *Apamea epomidion* | 3 |
| Noctuidae | *Apamea lateritia* | 8 |
| Noctuidae | *Apamea furva* | 8 |
| Noctuidae | *Apamea rubrirena* | 2 |
| Noctuidae | *Apamea oblonga* | 5 |
| Noctuidae | *Apamea remissa* | 7 |
| Noctuidae | *Apamea unanimis* | 5 |
| Noctuidae | *Apamea illyria* | 5 |
| Noctuidae | *Apamea anceps* | 5 |
| Noctuidae | *Apamea sordens* | 7 |
| Noctuidae | *Apamea scolopacina* | 8 |
| Noctuidae | *Apamea ophiogramma* | 8 |
| Noctuidae | *Eremobina pabulatricula* | 1 |
| Noctuidae | *Oligia strigilis* | 7 |
| Noctuidae | *Oligia versicolor* | 3 |
| Noctuidae | *Oligia latruncula* | 7 |
| Noctuidae | *Oligia fasciuncula* | 8 |
| Noctuidae | *Mesoligia furuncula* | 7 |
| Noctuidae | *Mesoligia literosa* | 6 |
| Noctuidae | *Mesapamea secalis* | 7 |
| Noctuidae | *Mesapamea didyma* | 5 |
| Noctuidae | *Photedes captiuncula* | 3 |
| Noctuidae | *Photedes minima* | 8 |
| Noctuidae | *Eremobia ochroleuca* | 6 |
| Noctuidae | *Luperina testacea* | 7 |
| Noctuidae | *Rhizedra lutosa* | 7 |
| Noctuidae | *Amphipoea oculea* | 7 |
| Noctuidae | *Amphipoea fucosa* | 7 |
| Noctuidae | *Amphipoea lucens* | 4 |
| Noctuidae | *Amphipoea crinanensis* | 5 |
| Noctuidae | *Hydraecia micacea* | 7 |
| Noctuidae | *Hydraecia ultima* | 5 |
| Noctuidae | *Hydraecia nordstroemi* | 6 |
| Noctuidae | *Hydraecia petasitis* | 3 |
| Noctuidae | *Gortyna flavago* | 6 |
| Noctuidae | *Calamia tridens* | 7 |
| Noctuidae | *Staurophora celsia* | 6 |
| Noctuidae | *Celaena haworthii* | 7 |
| Noctuidae | *Celaena leucostigma* | 8 |
| Noctuidae | *Nonagria typhae* | 7 |
| Noctuidae | *Phragmatiphila nexa* | 2 |
| Noctuidae | *Archanara geminipuncta* | 6 |
| Noctuidae | *Archanara dissoluta* | 6 |
| Noctuidae | *Archanara sparganii* | 6 |
| Noctuidae | *Archanara algae* | 6 |
| Noctuidae | *Sedina buettneri* | 4 |
| Noctuidae | *Arenostola phragmitidis* | 8 |
| Noctuidae | *Chortodes extrema* | 3 |
| Noctuidae | *Chortodes fluxa* | 7 |
| Noctuidae | *Chortodes pygmina* | 7 |
| Noctuidae | *Chortodes elymi* | 7 |
| Noctuidae | *Chortodes brevilinea* | 1 |
| Noctuidae | *Coenobia rufa* | 2 |
| Noctuidae | *Discestra trifolii* | 8 |
| Noctuidae | *Anarta myrtilli* | 6 |
| Noctuidae | *Anarta cordigera* | 3 |
| Noctuidae | *Lacanobia w-latinum* | 6 |
| Noctuidae | *Lacanobia oleracea* | 7 |
| Noctuidae | *Lacanobia thalassina* | 6 |
| Noctuidae | *Lacanobia contigua* | 6 |
| Noctuidae | *Lacanobia suasa* | 7 |
| Noctuidae | *Hada plebeja* | 6 |
| Noctuidae | *Aetheria dysodea* | 1 |
| Noctuidae | *Aetheria bicolorata* | 3 |
| Noctuidae | *Hadena bicruris* | 7 |
| Noctuidae | *Hadena compta* | 2 |
| Noctuidae | *Hadena confusa* | 4 |
| Noctuidae | *Hadena albimacula* | 4 |
| Noctuidae | *Hadena filograna* | 3 |
| Noctuidae | *Hadena caesia* | 1 |
| Noctuidae | *Hadena rivularis* | 6 |
| Noctuidae | *Hadena perplexa* | 5 |
| Noctuidae | *Hadena irregularis* | 3 |
| Noctuidae | *Sideridis albicolon* | 6 |
| Noctuidae | *Heliophobus reticulata* | 7 |
| Noctuidae | *Conisania leineri* | 1 |
| Noctuidae | *Melanchra persicariae* | 6 |
| Noctuidae | *Melanchra pisi* | 6 |
| Noctuidae | *Mamestra brassicae* | 6 |
| Noctuidae | *Papestra biren* | 5 |
| Noctuidae | *Polia bombycina* | 6 |
| Noctuidae | *Polia hepatica* | 6 |
| Noctuidae | *Polia nebulosa* | 7 |
| Noctuidae | *Mythimna turca* | 4 |
| Noctuidae | *Mythimna conigera* | 7 |
| Noctuidae | *Mythimna ferrago* | 6 |
| Noctuidae | *Mythimna albipuncta* | 4 |
| Noctuidae | *Mythimna pudorina* | 5 |
| Noctuidae | *Mythimna straminea* | 5 |
| Noctuidae | *Mythimna impura* | 7 |
| Noctuidae | *Mythimna pallens* | 7 |
| Noctuidae | *Mythimna obsoleta* | 7 |
| Noctuidae | *Mythimna comma* | 7 |
| Noctuidae | *Mythimna flammea* | 2 |
| Noctuidae | *Mythimna litoralis* | 2 |
| Noctuidae | *Mythimna l-album* | 4 |
| Noctuidae | *Orthosia incerta* | 5 |
| Noctuidae | *Orthosia gothica* | 6 |
| Noctuidae | *Orthosia cruda* | 5 |
| Noctuidae | *Orthosia miniosa* | 5 |
| Noctuidae | *Orthosia opima* | 6 |
| Noctuidae | *Orthosia populeti* | 3 |
| Noctuidae | *Orthosia cerasi* | 6 |
| Noctuidae | *Orthosia gracilis* | 6 |
| Noctuidae | *Orthosia munda* | 4 |
| Noctuidae | *Panolis flammea* | 7 |
| Noctuidae | *Cerapteryx graminis* | 8 |
| Noctuidae | *Tholera cespitis* | 8 |
| Noctuidae | *Tholera decimalis* | 8 |
| Noctuidae | *Pachetra sagittigera* | 4 |
| Noctuidae | *Eriopygodes imbecilla* | 1 |
| Noctuidae | *Lasionycta proxima* | 6 |
| Noctuidae | *Axylia putris* | 7 |
| Noctuidae | *Ochropleura plecta* | 7 |
| Noctuidae | *Diarsia mendica* | 6 |
| Noctuidae | *Diarsia dahlii* | 6 |
| Noctuidae | *Diarsia brunnea* | 6 |
| Noctuidae | *Diarsia rubi* | 8 |
| Noctuidae | *Diarsia florida* | 6 |
| Noctuidae | *Noctua pronuba* | 8 |
| Noctuidae | *Noctua orbona* | 8 |
| Noctuidae | *Noctua comes* | 8 |
| Noctuidae | *Noctua fimbriata* | 8 |
| Noctuidae | *Noctua janthina* | 6 |
| Noctuidae | *Noctua janthe* | 7 |
| Noctuidae | *Noctua interjecta* | 8 |
| Noctuidae | *Epilecta linogrisea* | 3 |
| Noctuidae | *Lycophotia porphyrea* | 7 |
| Noctuidae | *Chersotis cuprea* | 4 |
| Noctuidae | *Rhyacia simulans* | 7 |
| Noctuidae | *Epipsilia grisescens* | 4 |
| Noctuidae | *Standfussiana lucernea* | 4 |
| Noctuidae | *Paradiarsia glareosa* | 6 |
| Noctuidae | *Eurois occulta* | 8 |
| Noctuidae | *Spaelotis ravida* | 8 |
| Noctuidae | *Spaelotis clandestina* | 2 |
| Noctuidae | *Opigena polygona* | 4 |
| Noctuidae | *Graphiphora augur* | 5 |
| Noctuidae | *Eugnorisma depuncta* | 6 |
| Noctuidae | *Xestia speciosa* | 4 |
| Noctuidae | *Xestia alpicola* | 4 |
| Noctuidae | *Xestia c-nigrum* | 8 |
| Noctuidae | *Xestia ditrapezium* | 3 |
| Noctuidae | *Xestia triangulum* | 8 |
| Noctuidae | *Xestia ashworthii* | 3 |
| Noctuidae | *Xestia baja* | 8 |
| Noctuidae | *Xestia rhomboidea* | 5 |
| Noctuidae | *Xestia castanea* | 7 |
| Noctuidae | *Xestia sexstrigata* | 8 |
| Noctuidae | *Xestia xanthographa* | 8 |
| Noctuidae | *Eugraphe sigma* | 3 |
| Noctuidae | *Coenophila subrosea* | 5 |
| Noctuidae | *Cerastis rubricosa* | 6 |
| Noctuidae | *Cerastis leucographa* | 4 |
| Noctuidae | *Naenia typica* | 6 |
| Noctuidae | *Anaplectoides prasina* | 8 |
| Noctuidae | *Protolampra sobrina* | 6 |
| Noctuidae | *Actebia praecox* | 8 |
| Noctuidae | *Euxoa lidia* | 3 |
| Noctuidae | *Euxoa recussa* | 4 |
| Noctuidae | *Euxoa nigricans* | 8 |
| Noctuidae | *Euxoa tritici* | 7 |
| Noctuidae | *Euxoa obelisca* | 7 |
| Noctuidae | *Euxoa vitta* | 2 |
| Noctuidae | *Euxoa cursoria* | 7 |
| Noctuidae | *Agrotis ripae* | 7 |
| Noctuidae | *Agrotis exclamationis* | 8 |
| Noctuidae | *Agrotis clavis* | 8 |
| Noctuidae | *Agrotis segetum* | 8 |
| Noctuidae | *Agrotis vestigialis* | 8 |
| Noctuidae | *Agrotis cinerea* | 6 |
| Noctuidae | *Panthea coenobita* | 7 |
| Noctuidae | *Trichosea ludifica* | 1 |
| Noctuidae | *Colocasia coryli* | 6 |
| Others | *Lymantria monacha* | 6 |
| Others | *Lymantria dispar* | 4 |
| Others | *Calliteara pudibunda* | 7 |
| Others | *Calliteara abietis* | 3 |
| Others | *Dicallomera fascelina* | 5 |
| Others | *Orgyia recens* | 2 |
| Others | *Orgyia antiqua* | 7 |
| Others | *Orgyia antiquiodes* | 4 |
| Others | *Euproctis similis* | 7 |
| Others | *Leucoma salicis* | 7 |
| Others | *Arctornis l-nigrum* | 3 |
| Others | *Meganola strigula* | 5 |
| Others | *Meganola albula* | 5 |
| Others | *Nola cucullatella* | 7 |
| Others | *Nola confusalis* | 6 |
| Others | *Nola aerugula* | 6 |
| Others | *Nycteola revayana* | 7 |
| Others | *Nycteola degenerana* | 4 |
| Others | *Bena bicolorana* | 7 |
| Others | *Pseudoips prasinana* | 7 |
| Others | *Earias clorana* | 7 |
| Others | *Earias vernana* | 3 |
| Others | *Nudaria mundana* | 5 |
| Others | *Thumatha senex* | 8 |
| Others | *Miltochrista miniata* | 4 |
| Others | *Cybosia mesomella* | 7 |
| Others | *Pelosia muscerda* | 6 |
| Others | *Pelosia obtusa* | 5 |
| Others | *Atolmis rubricollis* | 7 |
| Others | *Lithosia quadra* | 6 |
| Others | *Eilema depressa* | 7 |
| Others | *Eilema lurideola* | 7 |
| Others | *Eilema complana* | 8 |
| Others | *Eilema pygmaeola* | 4 |
| Others | *Eilema lutarella* | 8 |
| Others | *Eilema sororcula* | 3 |
| Others | *Setina irrorella* | 5 |
| Others | *Setina roscida* | 2 |
| Others | *Dysauxes ancilla* | 1 |
| Others | *Spiris striata* | 2 |
| Others | *Coscinia cribraria* | 7 |
| Others | *Phragmatobia fuliginosa* | 8 |
| Others | *Parasemia plantaginis* | 3 |
| Others | *Spilosoma lutea* | 8 |
| Others | *Spilosoma lubricipeda* | 8 |
| Others | *Spilosoma urticae* | 6 |
| Others | *Diaphora mendica* | 7 |
| Others | *Diacrisia sannio* | 7 |
| Others | *Hyphoraia aulica* | 2 |
| Others | *Arctia caja* | 8 |
| Others | *Callimorpha dominula* | 6 |
| Others | *Tyria jacobaeae* | 6 |
|  |  |  |
